# Dissecting cell death pathways in fed‐batch bioreactors

**DOI:** 10.1002/biot.202300257

**Published:** 2023-12-07

**Authors:** David A. Mentlak, John Raven, Tessa Moses, Fraser Massie, Nicholas Barber, Robyn Hoare, Graeme Burton, Alison Young, Leon P. Pybus, Susan Rosser, Robert J. White, Daniel Ungar, Nia J. Bryant

**Affiliations:** ^1^ Department of Biology University of York, Heslington York UK; ^2^ FUJIFILM Diosynth Biotechnologies Mammalian Cell Culture Process Development Billingham UK; ^3^ EdinOmics RR*ID:SCR_021838 University of Edinburgh Max Born Crescent Edinburgh UK; ^4^ UK Centre for Mammalian Synthetic Biology School of Biological Sciences University of Edinburgh Edinburgh UK

**Keywords:** apoptosis, CHO cells, ferroptosis, industrial biotechnology, Parthanatos

## Abstract

Chinese hamster ovary (CHO) cells are widely used for production of biologics including therapeutic monoclonal antibodies. Cell death in CHO cells is a significant factor in biopharmaceutical production, impacting both product yield and quality. Apoptosis has previously been described as the major form of cell death occurring in CHO cells in bioreactors. However, these studies were undertaken when less was known about non‐apoptotic cell death pathways. Here, we report the occurrence of non‐apoptotic cell death in an industrial antibody‐producing CHO cell line during fed‐batch culture. Under standard conditions, crucial markers of apoptosis were not observed despite a decrease in viability towards the end of the culture; only by increasing stress within the system did we observe caspase activation indicative of apoptosis. In contrast, markers of parthanatos and ferroptosis were observed during standard fed‐batch culture, indicating that these non‐apoptotic cell death pathways contribute to viability loss under these conditions. These findings pave the way for targeting non‐conventional cell death pathways to improve viability and biologic production in CHO cells.

## Graphical Abstract and Lay Summary

Antibody‐producing CHO cells decline in viability towards the end of fed‐batch culture, which can have negative impacts on product yield and quality. Previous studies stated that apoptosis was the main cause of cell death. This study investigated more recently characterized non‐apoptotic forms of cell death during fed‐batch culture. Markers of apoptosis could not be observed under standard conditions. However, markers of two non‐apoptotic pathways, parthanatos and ferroptosis, were associated with viability loss. Only by increasing the impeller speed could markers of apoptosis be observed. These findings provide a framework to improve biologics production by targeting non‐apoptotic death pathways in CHO cells.

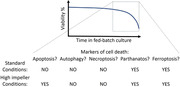

## 1 INTRODUCTION

Monoclonal antibody (mAb)‐based biologics represent the highest‐selling class of drugs in the biopharmaceutical industry, with sales of $217 billion dollars in 2021.^[^ ^1^ ^]^ Chinese Hamster Ovary (CHO) cells are responsible for the production of the majority of these drugs, as they provide numerous benefits including the capability of producing high‐titre products with the appropriate post‐translational modifications. Fed‐batch culture remains the dominant culture mode for CHO biopharmaceutical production.^[^ ^2^ ^]^ During fed‐batch culture, cells are supplied with additional feeds that replenish depleted nutrients. However, high‐viability cells cannot be maintained indefinitely during fed‐batch culture, as toxic metabolite accumulation and increased cellular stress ultimately lead to cell death.^[^ ^3^ ^]^

Cell death affects multiple aspects of antibody production and maintaining a viable cell population is fundamental to production. Protein quality can also be affected by cell viability, as cytolysis releases glycosidases and proteases that alter the N‐glycosylation pattern and degrade the secreted protein.^[^ ^4^, ^5^, ^6^ ^]^ Furthermore, cell death can cause difficulties during downstream processing due to the increase in cellular debris and host cell protein impurities.^[^ ^7^, ^8^ ^]^ Understanding the molecular pathways that control CHO cell death in bioreactors under fed‐batch conditions is therefore important to inform strategies that could increase viability and improve product quantity and quality.

Cell death can be broadly divided into two forms; accidental cell death and regulated cell death (RCD) (reviewed in ^[^ ^9^ ^]^). Accidental cell death occurs in response to severe physical, chemical or mechanical insults and leads to rapid unpreventable cell death. In contrast, during RCD specific molecular pathways are activated to initiate cell death. These pathways can be inhibited through genetic or pharmacological interventions, allowing the cell death response to be prevented or delayed. Apoptosis is the most well characterized form of RCD and leads to distinct cellular changes such as chromatin condensation, DNA laddering, cell shrinking, and cellular blebbing.^[^ ^10^ ^]^ Apoptosis occurs through two main pathways, the extrinsic and intrinsic pathways, that are activated by extracellular ligands or intracellular perturbations respectively.^[^ ^11^ ^]^ The early stages of both pathways involve activation of initiator caspases, caspase‐8 or caspase‐9.^[^ ^12^ ^]^ The pathways then converge on the activation of executioner caspases, such as caspase‐3 and caspase‐7, which bring about cellular destruction and demise.^[^ ^13^ ^]^

Previous studies have concluded that apoptosis is the main form of regulated cell death occurring in CHO cells under batch conditions.^[^ ^14^, ^15^, ^16^ ^]^ However, these studies were published prior to the expansion of knowledge in regulated cell death pathways over the past twenty years. The Nomenclature Committee on Cell Death recently defined ten non‐apoptotic death pathways with characteristic cellular and morphological hallmarks.^[^ ^11^ ^]^ Some of these pathways, such as necroptosis, culminate in plasma membrane disruption that is initiated by pore‐forming proteins.^[^ ^17^ ^]^ Non‐apoptotic RCD can also be initiated by alterations to the activity of key enzymes involved in cellular damage repair or prevention, as is observed in parthanatos and ferroptosis; hyperactivation of poly(ADP‐ribose) polymerase‐1 (PARP‐1) and lipid peroxidation respectively.^[^ ^18^ ^]^

Although RCD pathways are described as being distinct, there is interconnectivity between different death pathways,^[^ ^19^ ^]^ and non‐apoptotic cell death activation can sometimes culminate in caspase activation leading to apoptosis.^[^ ^20^, ^21^ ^]^ In addition, hallmarks that were previously thought to be unique to apoptotic cells, such as DNA fragmentation and phosphatidylserine plasma membrane exposure, can be observed during non‐apoptotic cell death.^[^ ^22^, ^23^, ^24^ ^]^ There is evidently a need to re‐evaluate cell death mechanisms in CHO cell cultures in light of the wider understanding of non‐apoptotic cell death and the links between various cell death pathways.

In this study, we examined apoptotic and non‐apoptotic cell death pathway activation in CHO cells during fed‐batch culture. We focused the majority of our studies on bioreactor conditions that represent standard operating conditions used by the biopharmaceutical industry (standard media and impeller speed) and found that apoptosis is not the main form of cell death occurring in these settings. We also found that biomarkers of two non‐apoptotic pathways, parthanatos and ferroptosis, are associated with the decline in viability. Apoptosis only became apparent in this system when stress was increased using higher impeller speed.

## 2 MATERIALS AND METHODS

### 2.1 Cell lines and culture maintenance

Two proprietary cell lines (A and B), developed by FUJIFILM Diosynth Biotechnologies, were used in this study. These cell lines were developed by transfecting the Apollo X host cell line (a derivative of CHO‐DG44; FUJIFILM Diosynth Biotechnologies), with expression plasmids for either an IgG1 (cell line A) or an IgG2 (cell line B) mAb, followed by clonal selection.^[^ ^25^ ^]^ Both cell lines were subcultured using proprietary FDB‐MAP medium (Sigma Aldrich). CHO‐S cells were used for apoptosis induction experiments and were subcultured using Freestyle CHO medium (Thermo Fisher Scientific) supplemented with 8 mM L‐glutamine. For routine subculture, all cell lines were incubated at 37°C, 5% CO_2_, on a shaking platform in 125 mL shake flasks and were subcultured every 3 to 4 days.

### 2.2 Bioreactor operation

Fed‐batch cultures were performed in 10‐L single‐use bioreactors using the Xcellerex XDR 10 (Cytiva) system. Cells were seeded into the bioreactor at an initial concentration of 0.5 × 10^6^ cells mL^−1^. The bioreactor basal growth medium was proprietary 514 (FUJIFILM Irvine Scientific) or MAP media, supplemented with 8 mM L‐glutamine. Cultures were maintained at 37°C, 40% dissolved oxygen, and pH was controlled through CO_2_ sparging and base additions. Nitrogen sparging was used to normalize total sparging across all bioreactors. Cultures were fed daily from day 2 with feed 7A (Cytiva), feed 7B (Cytiva) and glutamine, whereas glucose feeding was performed when the measured levels in the media fell below a threshold value. Cultures were agitated using a power per volume of 58.3 W m^−3^ (representing standard conditions), or 526.7 W m^−3^ to test the effect of high‐impeller speeds on cell‐death processes. ADCF Antifoam agent (Hyclone) was added to control foaming as necessary. Samples were collected daily for cell counting, metabolite analysis and western blot analysis.

### 2.3 Bioprocess analytical methods

Viable cell counts and viability were assessed by the trypan blue exclusion assay using a ViCell XR Cell Viability Analyzer (Beckman Coulter). Glucose, glutamine, lactate, and ammonium were measured using a BioProfile FLEX2 (Nova Biomedical). Osmolality was analyzed using an Advanced 3320 Micro‐Osmometer (Advanced Instruments). Dissolved oxygen was measured using a RAPIDLab 348EX blood gas system (Siemens).

### 2.4 Western blotting

Daily samples were removed from the bioreactors and centrifuged at 1000 × *g* for 5 min, and the pellet was then frozen at −80°C for later analysis. Cell pellets were lysed and boiled in SDS‐PAGE sample buffer (150 mM Tris pH 6.8, 8% w/v SDS, 166 mM DTT) for 5 min at 97°C, followed by protein separation using SDS‐PAGE gel electrophoresis. Resolved proteins were transferred to nitrocellulose or PVDF membranes, using either semi‐dry transfer (Bio‐Rad) or an iBlot 2 Dry Blotting System (ThermoFisher Scientific), according to the manufacturer's guidelines. Membranes were blocked at room‐temperature for 1 h in 5% (w/v) milk powder in TBS‐T or PBS‐T, or 10% (v/v) blocking solution (Roche) in TBS‐T. Primary antibodies were incubated overnight at 4°C in the blocking buffer. After being washed six times with TBS‐T or PBS‐T, the membranes were incubated for 1 h at room temperature with the HRP‐conjugated secondary antibody, diluted in blocking buffer. The membranes were washed a further six times and were then imaged using an iBright Imaging System (Thermo Fisher Scientific) after incubation with the chemiluminescent substrate, BM Chemiluminescence Western Blotting Substrate (Roche) or SuperSignal West Pico Plus Chemiluminescent Substrate (Thermo Fisher Scientific). To ensure the linearity of the signal, a dilution series of one sample was included on technical replicates.

### 2.5 Antibodies

The following antibodies were used in this study: anti‐caspase‐9 (CST, 9508S, 1:1000), anti‐caspase‐3 (CST, 14220S, 1:500), anti‐cleaved caspase‐3 (CST, 9664S, 1:500), anti‐PARP (CST, 9542S, 1:1000), anti‐LC3 (Nanotools, 0231‐100, 1:1000), anti‐phosphorylated MLKL (S345; abcam, ab196436, 1:200), anti‐MLKL (Proteintech, 21066‐1‐AP, 1:200), anti‐PAR (Enzo, ALX‐804‐220‐R100, 1:5000), anti‐GPX4 (abcam, ab125066, 1:1000), anti‐FSP1 (Proteintech, 20886‐1‐AP, 1:500), anti‐MDA (abcam, ab6463, 1:500), anti‐GPX1 (abcam, ab108427, 1:100), anti‐SOD1 (Santa Cruz, sc‐101523, 1:2000), anti‐SOD2 (Proteintech, 24127‐1‐AP, 1:2000), anti‐rabbit HRP (CST, 7074S, 1:2000), anti‐mouse HRP (Jackson, 115‐035‐146, 1:2000).

### 2.6 Western blot analysis

Western blot images were quantified by densitometry using Fiji,^[^ ^26^ ^]^ according to recommended guidelines ^[^ ^27^ ^]^ using the “Gels” function. Firstly, the rectangle tool was used to select all lanes and plot the profile areas. The band peak was then isolated above the background level using the straight‐line tool. Finally, the wand tool was used to calculate the area of the peak in arbitrary units. Target protein band densities were normalised to the signal of a selected day (specified in the figure legends) and the total protein staining of the sample derived from quantifying a full lane of a Coomassie brilliant blue stained SDS‐PAGE gel. Average normalised values were derived from the quantification of two technical replicates of the western blot unless stated otherwise.

### 2.7 Mass spectrometry analysis of malondialdehyde (MDA)

MDA standard was prepared by dissolving 25 μL 1,1,3,3 tetraethoxypropane (TEP, Sigma‐Aldrich T9889‐25ML) in 100 mL of water to give a 1 mM stock solution. Working standard of MDA was prepared by hydrolysis of 1 mL TEP stock solution in 50 mL 1% sulfuric acid and incubation for 2 h at room temperature.

Metabolites were extracted from CHO cell pellets using a fixed ratio of cell number to extraction solvent volume. A volume of 500 μL chloroform/methanol/water (1/3/1 ratio) was added to 40 × 10^6^ cells and mixed vigorously at 1000 rpm for 1 h at 4°C. The extraction mixtures were then centrifuged at 13,000 × *g* for 10 min, and 100 μL of the supernatant was transferred into sterile 1.5 mL microcentrifuge tubes for storage at −80°C until analysis.

The targeted metabolomics analysis was performed using liquid chromatography (LC) coupled to ion mobility (IM) quadrupole time of flight (qTOF) mass spectrometry (MS) as described previously.^[^ ^28^ ^]^ The instrumentation consisted of an Agilent 1290 Infinity II series UHPLC system hyphenated with an Agilent 6560 IM‐qTOF with a Dual Agilent Jet Stream Electron Ionization source. In brief, LC separation was performed on an InfinityLab Poroshell 120 HILIC‐Z, 2.1 mm × 50 mm, 2.7 μm UHPLC column (Agilent Technologies 689775–924) coupled to an InfinityLab Poroshell 120 HILIC‐Z, 3.0 mm × 2.7 μm UHPLC guard column (Agilent Technologies 823750–948). A 3.5 min gradient was run using organic buffer (acetonitrile) combined with an aqueous buffer with low pH (10 mM ammonium formate, pH 3) or high pH (10 mM ammonium acetate, pH 9) for positive and negative ionization modes, respectively. For analysis, 1 μL of sample was injected into the column. A pooled quality control sample was generated by combining equal volumes of each sample and injected five times at the beginning of the experiment to condition the column and after every five samples to monitor the instrument state over the course of data acquisition. Data were acquired in the 50 to 1700 *m/z* range, with an MS acquisition rate of 0.8 scans/s. Data from three technical replicates were acquired for each sample.

### 2.8 Apoptosis induction

Exponentially growing CHO‐S cells were seeded at 1 × 10^6^ cells mL^−1^, in 125 mL flasks with a 20 mL working volume. Apoptosis was induced with the addition of 2 μM staurosporine (10 mM stock in dimethylsulfoxide (DMSO)), or the addition of a DMSO vehicle control, and the cells were incubated at 37°C, 5% CO_2_, on a shaking platform for 24 h. Viability was assessed using a ViCell XR, and cell lysates were prepared as described previously.

## 3 RESULTS

### 3.1 Apoptosis is not the cause of decreased cell viability in a CHO fed‐batch bioreactor

Apoptosis has previously been reported as the predominant form of cell death in CHO cells grown in bioreactors under batch and fed‐batch conditions.^[^ ^15^, ^29^ ^]^ However, advances in our understanding of non‐apoptotic cell death necessitate a re‐evaluation of cell death in an industrial setting. To this end, a high‐producing proprietary CHO cell line (referred to as line A) was grown under fed‐batch conditions over a 14‐day culture period using a 10‐L stirred‐tank bioreactor. Viable cell growth peaked in this cell line at day 12; however, cell viability gradually decreased from day 4 to day 14 (Figure S1).

**FIGURE 1:**
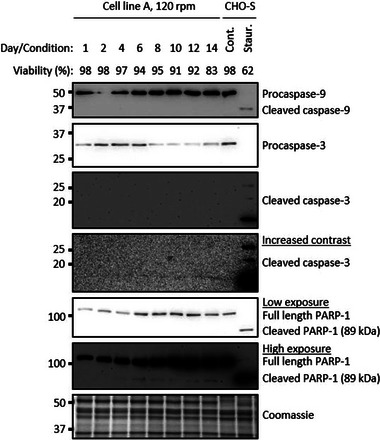
**Apoptosis is not evident during fed‐batch culture**. Western blot analysis of apoptotic cleaved caspases and PARP‐1 during fed‐batch bioreactor cultures of cell line A. As a positive control for apoptosis, CHO‐S cells were treated with 2 μM staurosporine for 24 h. Whole cell lysate was separated using SDS‐PAGE gel electrophoresis. Cont. = DMSO vehicle control, Staur. = 24 h 2 μM staurosporine treatment.

To determine the molecular pathways underpinning the observed reduction in cell viability, markers of various death pathways were analyzed. Three apoptosis markers were investigated; the initiator caspase, caspase‐9, an executioner caspase, caspase‐3, and the downstream target of active caspase‐3, PARP‐1 (Figure 1 and Figure S2A). Cleaved forms of the activated caspases and PARP‐1 were detected in staurosporine treated CHO‐S cells, a positive control for induction of apoptosis. However, cleaved caspases could not be detected during the culture of cell line A (Figure 1). From day 6 to 14, a very weak cleaved PARP‐1 signal could be detected, however this signal did not increase proportionally with the decrease in viability. These results suggest that apoptosis does not account for the loss of viability in cell line A, under the fed‐batch bioreactor conditions used.

As these findings challenge the widely held view that apoptosis is the predominant form of cell death when CHO cells are grown in bioreactors, we investigated whether this observation was specific to the culture medium and cell line used. When cell line A was cultured under identical conditions but using a different culture medium, markers of apoptosis were again absent (Figure S2C), despite a significant decline in cell viability towards the end of the culture period. When a different proprietary cell line was grown under these conditions, a very weak cleaved caspase‐3 and cleaved PARP‐1 signal could be detected from day 4, but this signal did not increase as the cell viability declined (Figure S2E). These results suggest that under standard industrial fed‐batch conditions apoptosis is not primarily responsible for the decline in cell viability, observations that are not restricted to an individual cell line or medium.

We reasoned that an alternative non‐apoptotic regulated cell death pathway may be responsible for cell death in the fed‐batch cultures being studied here. Of the ten non‐apoptotic pathways characterized in mammalian cells,^[^ ^11^ ^]^ we ruled out those unlikely to be relevant to a CHO cell: namely immunogenic cell death (ICD) and NETotic (“Neutrophil Extracellular Trap”‐otic) cell death, as ICD requires immune cells to initiate death and NETotic cell death is restricted to hematopoietic cells.^[^ ^11^ ^]^ Entosis was also excluded, as this pathway describes cell death of an engulfed cell and would therefore not account for the viability losses as determined by the trypan blue exclusion assay. Pyroptosis was excluded as it has been primarily associated with pathogen and viral infection.^[^ ^30^ ^]^ Lysosome‐dependent cell death and mitochondrial permeability transition‐driven necrosis were also not investigated in this study due to the limited availability of biomarkers for these pathways. This focused our analyses on four non‐apoptotic cell death pathways; autophagy‐dependent cell death, necroptosis, parthanatos and ferroptosis.

### 3.2 Autophagy and necroptosis do not contribute substantially to CHO cell death under standard conditions

**FIGURE 2:**
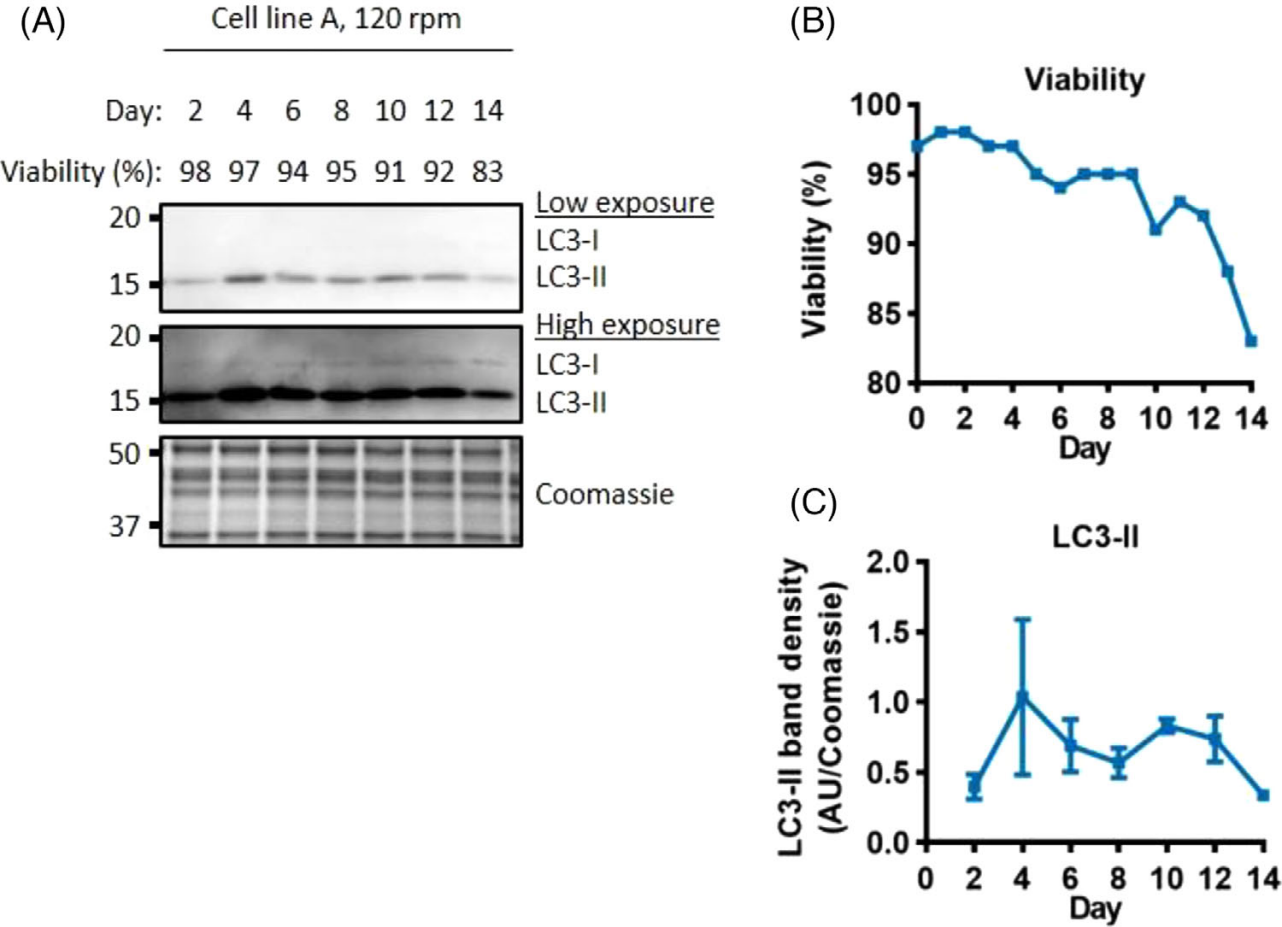
**Autophagy does not increase towards the end of the culture**. Western blot analysis of the autophagosome marker LC3‐II (A). Viability through the fed‐batch culture (B). Quantification of LC3‐II (C), error bars represent ± SD of two technical replicates, western blot signals on each day were normalised to the day 10 value. Whole cell lysate was separated using SDS‐PAGE gel electrophoresis.

Autophagy can function as a survival mechanism during nutrient deprivation and other stresses but can promote cell death in certain circumstances.^[^ ^31^ ^]^ Several reports have shown that autophagy occurs in CHO cells during batch and fed‐batch culture, and under stress conditions.^[^ ^29^, ^32^, ^33^ ^]^ Autophagy is frequently studied by examining the levels of the autophagosome‐binding protein microtubule‐associated protein light chain 3 (LC3). Cytosolic LC3 is cleaved shortly after synthesis to form LC3‐I, which can then be subsequently conjugated with phosphatidylethanolamine, to form LC3‐II. In this lipidated form, LC3‐II associates with the inner and outer membranes of the autophagosome.^[^ ^34^ ^]^ The accumulation of autophagy‐induced LC3‐II therefore serves as a marker of autophagy.^[^ ^35^ ^]^ The level of this marker was investigated in cell line A when cultured at the standard impeller speed using the 514 media (Figure 2 and Figure S3A), the same conditions under which apoptosis was not detected (Figure 1). LC3‐II was the predominant signal and only a weak LC3‐I signal was observed. LC3‐II could be detected at every time point tested, but showed a slight reduction in signal towards the end of the culture. While autophagy is thus present throughout the culture, its decrease when viability declines suggests that autophagy is unlikely to be responsible for the eventual viability decrease in cell line A.

**FIGURE 3:**
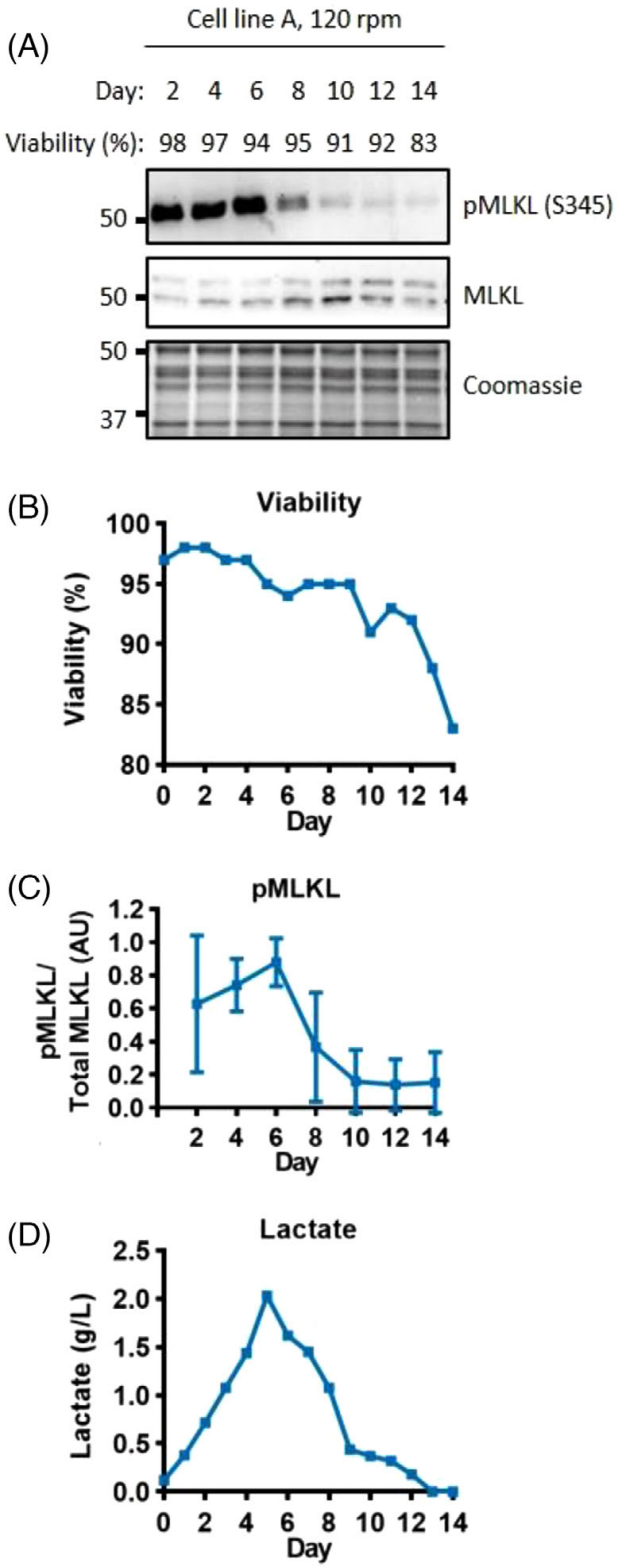
**Necroptosis markers correlate with the level of lactate**. Western blot analysis of the necroptosis marker pMLKL (A). Viability through the fed‐batch culture (B). Quantification of the level of pMLKL (C), error bars represent ± SD of two technical replicates, western blot signals on each day were normalised to the day 6 value. Lactate levels measured in the media (D). Whole cell lysate was separated using SDS‐PAGE gel electrophoresis.

We hypothesized that necroptosis could be occurring, as it can share hallmarks with apoptosis such as phosphatidylserine exposure on the outer plasma membrane and DNA laddering.^[^ ^23^, ^24^ ^]^ These shared features could have led to mischaracterization of apoptosis in previous CHO cell culture studies. Necroptosis is a form of regulated cell death mediated through the activation of mixed lineage kinase domain like pseudokinase (MLKL) by receptor interacting serine/threonine kinase 3 (RIPK3), under conditions of caspase‐8 inactivity.^[^ ^11^ ^]^ Activation of MLKL occurs through RIPK3‐mediated phosphorylation at serine 345 in murine models.^[^ ^36^ ^]^ This modification is critical for MLKL oligomerization, translocation to the plasma membrane and execution of necroptosis.^[^ ^36^, ^37^, ^38^ ^]^ The serine 345 site is conserved in CHO cells and could therefore be used to determine the level of activated MLKL by comparing the ratio of phosphorylated MLKL (pMLKL) to total MLKL. In our settings, the MLKL antibody recognized two bands at approximately 50 kDa. As these could represent two isoforms, both were included in the quantification of total MLKL. Figure 3 shows the level of this necroptotic marker throughout the culture period. The ratio of active pMLKL:MLKL was highest within the first six days of culture and then decreased dramatically towards the end of the culture period (Figure 3A and C, and Figure S4A). Comparing the levels of active pMLKL to the bioreactor metabolites and process outcomes, suggested that there could be a link between the level of active pMLKL and the level of lactate in the media. Lactate production increased from the start of the culture and peaked at day 5 before switching to lactate consumption, and all lactate was consumed by day 14 (Figure 3D). The level of pMLKL in the cells correlates with the increasing level of extracellular lactate and similar observations were made under different experimental conditions (Figure S4). However, this observation was not followed up further, as the peak of active pMLKL did not correlate with a loss in cell viability, which suggests that necroptosis does not contribute substantially to cell death under these conditions.

### 3.3 Parthanatos and ferroptosis biomarkers increase as the viability declines

**FIGURE 4:**
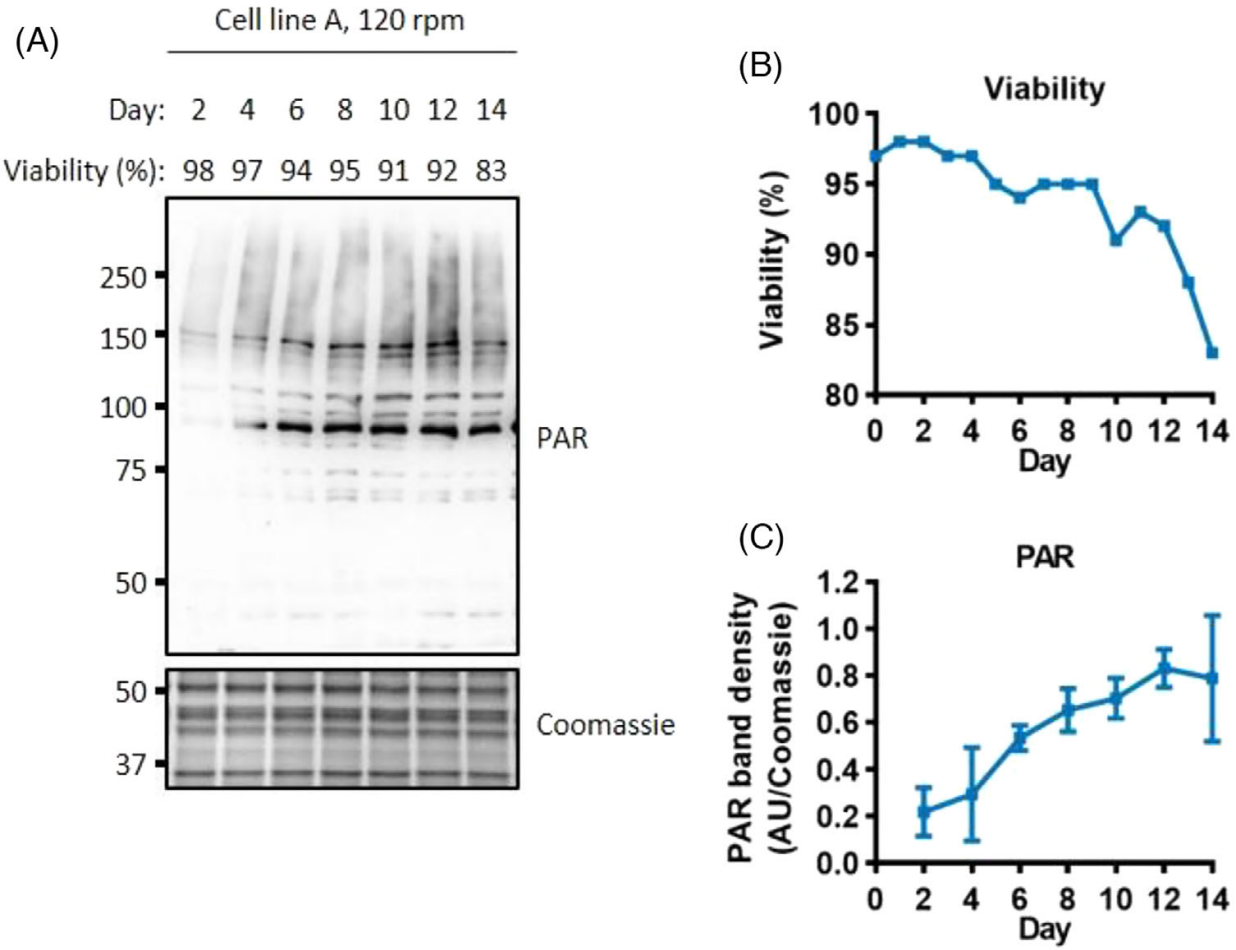
**PAR accumulation associated with a decrease in viability**. Western blot analysis of the parthanatos marker PAR (A). Viability through the fed‐batch culture (B). Quantification of the level of PAR (C), error bars represent ± SD of two technical replicates, western blot signals on each day were normalised to the day 12 value. Whole cell lysate was separated using SDS‐PAGE gel electrophoresis.

The cell death pathways tested so far did not account for the decline in viability in the bioreactor; we therefore investigated involvement of parthanatos and ferroptosis. Parthanatos is characterized by hyperactivation of PARP‐1, that can be caused by DNA alkylating agents as well as oxidative damage.^[^ ^11^ ^]^ The hyperactivation of this enzyme causes an accumulation of poly(ADP‐ribose) (PAR) polymers.^[^ ^39^, ^40^ ^]^ which cause the translocation of apoptosis inducing factor (AIF) from the mitochondria to the nucleus.^[^ ^21^, ^40^, ^41^ ^]^ where it can cause large scale DNA fragmentation and cell death.^[^ ^41^, ^42^ ^]^ As well as being toxic to the cell,^[^ ^39^ ^]^ PAR polymer production can also deplete the cell of ATP and NAD+, leading to a collapse in cellular energy, which further exacerbates cell death.^[^ ^43^, ^44^ ^]^ Figure 4 shows the accumulation of PAR during the fed‐batch culture. PAR accumulation increased substantially from days 2 to 4, and then showed a gradual increase up to day 12 before slightly dropping again on day 14 (Figure 4 and Figure S5A). As PAR accumulation increased as the viability dropped, parthanatos could contribute to cell death in cell line A.

**FIGURE 5:**
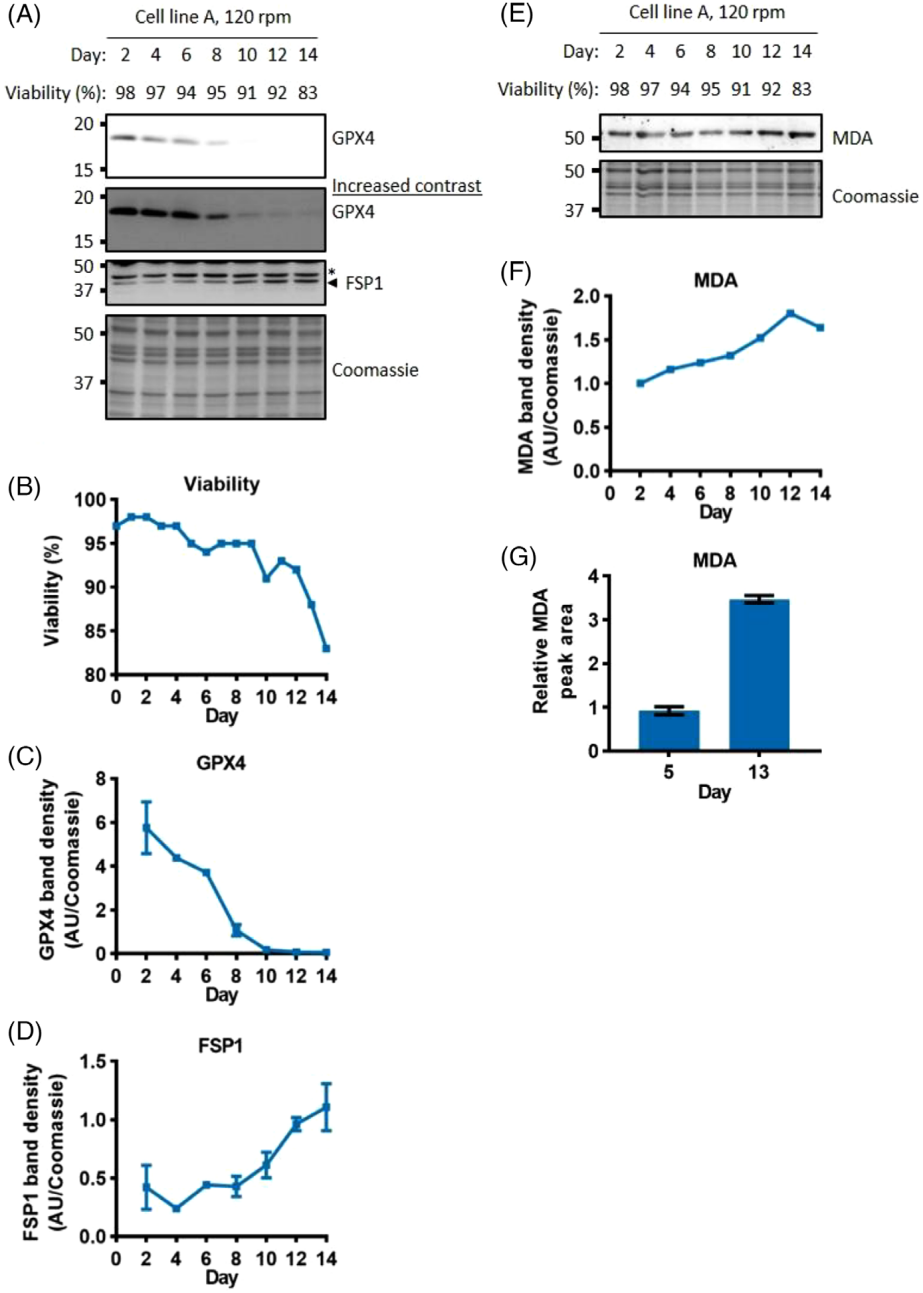
**Markers of ferroptosis are associated with a decline in viability**. Western blot analysis of ferroptosis suppression enzymes, GPX4 and FSP1 (A). Viability through the fed‐batch culture (B). Quantification of GPX4 and FSP1 (C, D), error bars represent ± SD of two technical replicates, western blot signals on each day were normalised to the day 8 and day 14 values respectively. Western blot analysis and quantification (due to discontinuation of the antibody only one technical replicate was performed) of lipid peroxidation marker MDA (E, F). Quantification of MDA using LC‐MS (G), error bars represent ± SD of three technical replicates. Whole cell lysate was separated using SDS‐PAGE gel electrophoresis. * = Non‐specific band.

Ferroptosis is driven by accumulation of toxic lipid peroxides.^[^ ^11^, ^45^ ^]^ The level of lipid peroxidation, and therefore sensitivity to ferroptosis, is controlled by several key enzymes including Glutathione Peroxidase 4 (GPX4), and the more recently identified Ferroptosis Suppressor Protein 1 (FSP1).^[^ ^46^, ^47^ ^]^ We first examined whether any sensitivity to ferroptosis could be present in the bioreactors by examining the level of these enzymes. Figure 5A shows that the level of GPX4, the canonical ferroptosis suppressor, is greatly diminished from day 10 of the culture. However, the level of FSP1 increased concurrently with the reduction of GPX4 reduction (Figure 5A and Figure S6). Similar changes were also observed for the antioxidant enzymes that are not involved in protection against lipid peroxides (Figure S8). Glutathione Peroxidase 1 (GPX1), like GPX4, showed a reduction towards the end of the culture, whereas the levels of Superoxide Dismutase 1 (SOD1) and SOD2 increased.

Given the observed shifts in ratio of GPX4 and FSP1, we sought to determine the overall impact on the level of lipid peroxidation. We studied progression of ferroptosis in the bioreactors by examining the level of MDA, a toxic product of lipid peroxidation that can form adducts onto cellular proteins.^[^ ^48^ ^]^ MDA levels increased towards the end of the culture, observed using western blots and LC‐MS (Figure 5E‐G), suggesting that ferroptosis is occurring and may cause the observed loss of viability.

### 3.4 High impeller speeds activate caspases

**FIGURE 6:**
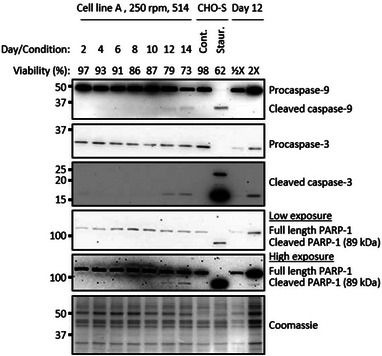
**Increased impeller speed leads to apoptosis activation**. Western blot analysis of apoptotic cleaved caspases and PARP‐1 during fed‐batch bioreactor cultures of cell line A at high impeller speeds. Whole cell lysate was separated using SDS‐PAGE gel electrophoresis. Cont. = DMSO vehicle control, Staur. = 24 h 2 μM staurosporine treatment.

Our results so far have demonstrated that apoptosis does not occur under standard industrial growth conditions. However, previous studies have shown that apoptosis can occur under batch and fed‐batch conditions.^[^ ^29^, ^49^ ^]^ We therefore tested whether apoptosis could be induced through increased stress. To increase stress on the cells, we increased impeller speed. Cell growth and viability were lower than the standard conditions under these conditions (Supp. Figure S1). Using higher impeller speed to culture cell line A, cleaved caspase‐9, −3 and PARP‐1 were all detected on days 12 and 14, when the viability dropped below 80% (Figure 6 and Figure S2B). However, the signal was considerably weaker in comparison to the staurosporine‐treated cells. This demonstrates that cell line A is capable of undergoing apoptosis under elevated stress conditions.

Having established that high impeller speed can induce apoptosis, we investigated whether it also impacted on the other forms of cell death we had studied. The progression of the autophagy marker LC3‐II at the high impeller speed was very similar to that under normal conditions (Figure S3 B and G). The necroptotic marker pMLKL also matched the observations made under standard conditions, with a strong signal being detected early on in the culture which corresponded to an increase in the lactate levels in the medium (Figure S4 B, E, H). In addition, there was another increase in active pMLKL at day 14, which was matched by an increase in lactate production. In light of this correlation, it is unclear whether the increased active pMLKL on day 14 of the high‐speed cultures contributes to cell death or is a reflection of this metabolic shift. Interestingly, the levels of PAR filaments increased dramatically as the viability dropped (Figure S5 B & G). The lipid peroxidation marker, MDA, also showed a large increase associated with the decline in cell viability (Figure S7 E, J, L). The data indicate that apoptosis, parthanatos, and ferroptosis are all activated at high impeller speeds and may contribute together to a loss of cell viability.

## 4 DISCUSSION

In contrast to previous findings, this study demonstrates that apoptosis is not always the major determinant of cell death in fed‐batch CHO cell cultures. Stirred tank 10‐litre bioreactors, under standard impeller speeds (P/V = 58.3 W m^−3^), showed a decline in viability without any detectable increase in cleaved caspases or cleaved caspase targets (Figure 1 and Figure S2). Although apoptosis has been the focus of cell death research in CHO cells, there have been fed‐batch studies that show a decline in viability with no increase in active caspases.^[^ ^29^, ^50^ ^]^ However, the authors of these studies did not further investigate the activation of non‐apoptotic cell death pathways occurring in their settings. Our results highlight that CHO cell death pathway activation is not limited to apoptosis.

Genetic engineering strategies aimed at prolonging viable cell growth in CHO cells have so far focused on the apoptotic pathway. Altering the levels of Bcl‐2 family proteins, caspases, and Inhibitor of Apoptosis Proteins (IAPs) has generally led to improvements in culture performance.^[^ ^3^, ^13^ ^]^ Although many of these proteins have clearly defined roles in the apoptotic pathway, they can also have roles in metabolism and non‐apoptotic cell death. Engineered anti‐apoptotic cells can exhibit shifts in lactate metabolism,^[^ ^51^, ^52^, ^53^, ^54^ ^]^ leading to an overall reduction in this growth‐inhibitory metabolite.^[^ ^55^, ^56^ ^]^ Regulators of the apoptotic pathway have also been shown to be involved in non‐apoptotic cell death pathways such as mitochondrial permeability transition driven necrosis, pyroptosis and necroptosis.^[^ ^57^, ^58^ ^]^ The previous success of “anti‐apoptotic” genetic engineering does not therefore contradict our results, as such alterations can have multiple secondary effects that could lead to an improvement in culture performance.

This study is the first to investigate the occurrence of non‐apoptotic death pathways in CHO cell bioreactors. Under standard conditions in cell line A, we were able to observe an increase in a parthanatos marker and changes indicative of ferroptosis (Table 1). Ferroptosis, which results in an accumulation of lipid peroxidation, and parthanatos, which results in PAR accumulation and large‐scale DNA fragmentation, have largely been studied independently. However, previous reports have demonstrated crossover between these two pathways; knocking‐down AIF can prevent erastin‐induced ferroptosis ^[^ ^59^ ^]^ and parthanatos can be initiated by reduced levels of GPX4.^[^ ^60^, ^61^ ^]^ One of the commonalities between these two pathways is the central role that reactive oxygen species (ROS) have in contributing towards death. ROS can contribute to the hyper‐activation of PARP‐1 during parthanatos, as well as causing lipid peroxidation during ferroptosis.^[^ ^11^, ^62^, ^63^ ^]^ A time‐dependent increase in oxidative stress was observed during this study, as evidenced by the increase in antioxidant enzymes (Supp Figure S8), which could therefore be the trigger of these non‐apoptotic death pathways.

**TABLE 1:** **Summary of cell death modalities in the bioreactors**. Notes: 1. Cleaved 89‐kDa PARP‐1 present as a small proportion of the total and no increase observed from day 6 to 14 as viability decreases.

Cell type	A				B	
Media	514		MAP		514	
Impeller speed	120	250	120	250	120	250
Day 10 and 14 viability (%)	91, 83	87, 79	92, 77	79, 70	93, 71	90, 86
Apoptosis (cleaved caspase‐9, −3, cleaved PARP)	Not detected ^1^	Weak cleaved caspase‐9, −3 and cleaved PARP signal on days 12 & 14 (viability < 80%)	Not detected. PARP not tested	Weak cleaved caspase‐9 on days 12 and 14, weak cleaved caspase‐3 on days 8–14, weak cleaved PARP on days 10–14	Not detected ^1^	Not detected ^1^
Autophagy (LC3‐II)	Peak levels did not correlate with lowest viabilities	Peak levels did not correlate with lowest viabilities	Detected from day 8, with a strong increase to day 14.	Strong increase on days 10–14.	Highest signal on days 12 and 14	Increased strongly towards day 14
Necroptosis (pMLKL)	Highest levels at day 1–6, and then drops off significantly	Increases from days 4 to 8 and drops back down, followed by another peak at day 14	Not tested	Not tested	Highest levels at day 1–6, and then drops off significantly	Increases from days 1 to 6 and drops back down, followed by another peak at day 14
Parthanatos (PAR)	Increases from day 1–12, and then slightly decreases.	Very strong increase towards the end of the culture, associated with a loss in viability	Increases from day 1–10, and then slightly decreases	Increases towards the end of culture, but peak levels observed on days 8 and 10 (viability, 96 and 79% respectively)	Increase from day 2 to 4, associated with a 5% drop in viability, but then plateaus	Increase from day 2 to 4, associated with a 4% drop in viability, but then plateaus
Ferroptosis (MDA)	Modest increase towards the end of the culture	Strong increase towards the end of culture	Little change across the culture period	Strong increase towards the end of culture	Little change across the culture period	Strong increase towards the end of culture
Ferroptosis suppression enzymes (GPX4, FSP1)	GPX4 decrease and FSP1 increase towards the end of the culture	Not tested	Not tested	Not tested	GPX4 decrease and FSP1 increase towards the end of the culture	Not tested

In this study, we only detected markers of apoptosis in the high impeller speed bioreactors (Figure 6 and Figure S2), as predicted by the observation that increased shear stress in HEK293 cells increases mRNA levels of apoptosis‐related genes.^[^ ^64^ ^]^ At this higher speed, the cells were subject to greater stress, as evidenced by the reduced growth and a lower viability throughout the culture (Figure S1). Metabolism was also affected and the high impeller bioreactor exhibited a slower transition to lactate consumption, which consequently required greater base additions to maintain the pH setpoint, leading to a culture with higher osmolality (Figure S9). Furthermore, increased anti‐foam additions were required to combat the greater extent of foaming that occurred under these conditions (Figure S10). Both the amount of foam and antifoam itself can negatively affect the gas exchange within the bioreactor,^[^ ^65^, ^66^ ^]^ which could therefore lead to hypoxic conditions. High osmolality and hypoxia have been shown to negatively affect cell growth and can lead to apoptosis activation.^[^ ^32^, ^67^, ^68^ ^]^ It is likely that the combination of these stresses, and perhaps other unknown stresses, contributed to the activation of apoptosis in this setting. In this study, apoptotic cell death was only induced under exaggerated stress conditions. These data suggest that previous reports of apoptosis in CHO cell cultures may have resulted from suboptimal process conditions.

In addition to cleaved caspases, non‐apoptotic markers of parthanatos and ferroptosis also increased as the viability declined in the high impeller bioreactors (Figure S5 and S7). Increases in lipid peroxidation have also recently been observed alongside markers of UPR‐driven apoptosis during fed‐batch CHO cell cultures.^[^ ^69^ ^]^ It is possible that the observed cleaved caspases in the high‐impeller speed bioreactors represent a final stage of cell death rather than the original triggering pathway. Alternatively, low level lipid peroxidation and PAR polymer production could be occurring alongside apoptosis in this setting, as has been hypothesized previously.^[^ ^70^ ^]^

## 5 CONCLUSION

This study demonstrates that apoptosis is not always the major cause of cell death in CHO cells grown in fed‐batch culture. Instead, markers of two non‐apoptotic pathways, parthanatos and ferroptosis, correlated with a decline in viability in these cultures. This discovery raises the possibility that targeting these pathways could improve cell growth characteristics, antibody titre and product quality.

## AUTHOR CONTRIBUTIONS

Conceptualisation: Nia J. Bryant, Daniel Ungar, Robert J. White; Investigation: David A. Mentlak, John Raven, Tessa Moses, Fraser Massie, Nicholas Barber, Robyn Hoare, Graeme Burton; Data curation: John Raven; Data analysis & interpretation: David A. Mentlak, Nia J. Bryant, Daniel Ungar, Robert J. White; Writing—original draft: David A. Mentlak; Writing—review & editing: Nia J. Bryant (equal), Daniel Ungar (equal), Robert J. White (equal), all other authors supported; Supervision: Nia J. Bryant, Daniel Ungar, Robert J. White, Alison Young, Leon P. Pybus, Nicholas Barber, Tessa Moses; Project administration: Nia J. Bryant, Daniel Ungar, Robert J. White, Alison Young, Leon P. Pybus; Funding acquisition: Susan Rosser, Nia J. Bryant, Daniel Ungar, Robert J. White.

## CONFLICT OF INTEREST STATEMENT

The authors declare no conflicts of interest.

## Supporting information

Supporting information

## Data Availability

The data that support the findings of this study are available from the corresponding author upon reasonable request.
