# Supplementary material for: Dissecting cell death pathways in fed‐batch bioreactors
Source: Biotechnol J. 2023 Dec 7;19(1):2300257. doi: 10.1002/biot.202300257 (PMC11475371; doi:10.1002/biot.202300257)
Supplement: Supplementary file 1 — Supporting information [file BIOT-19-2300257-s001.pdf]

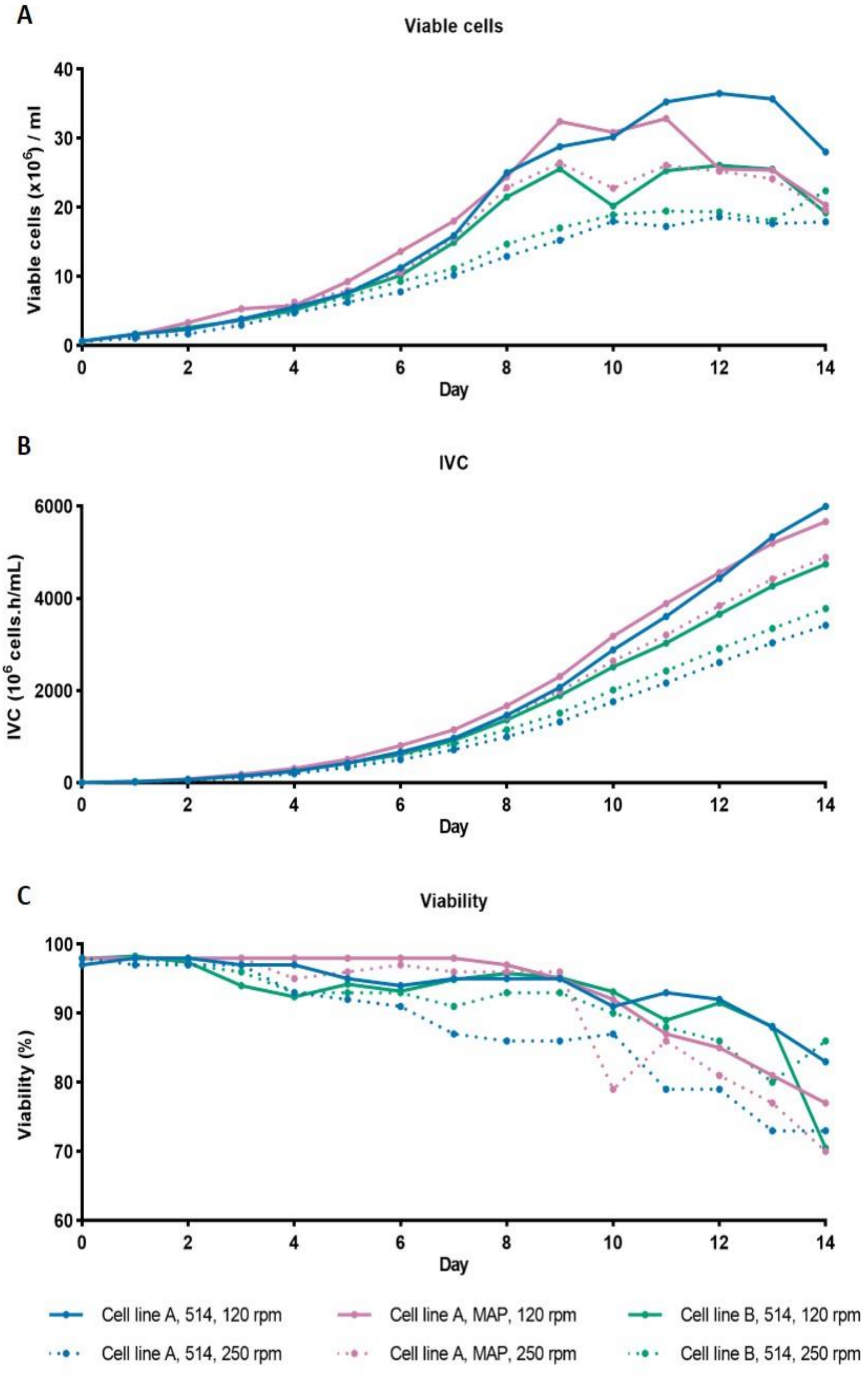

Supplementary Figure S1: Increased impeller speed affects cellular growth and viability. Profile of cell characteristics of cells grown in 10 litre bioreactors under fed-batch conditions. Viable cell growth (A), integral of viable cells (B), viability (C).

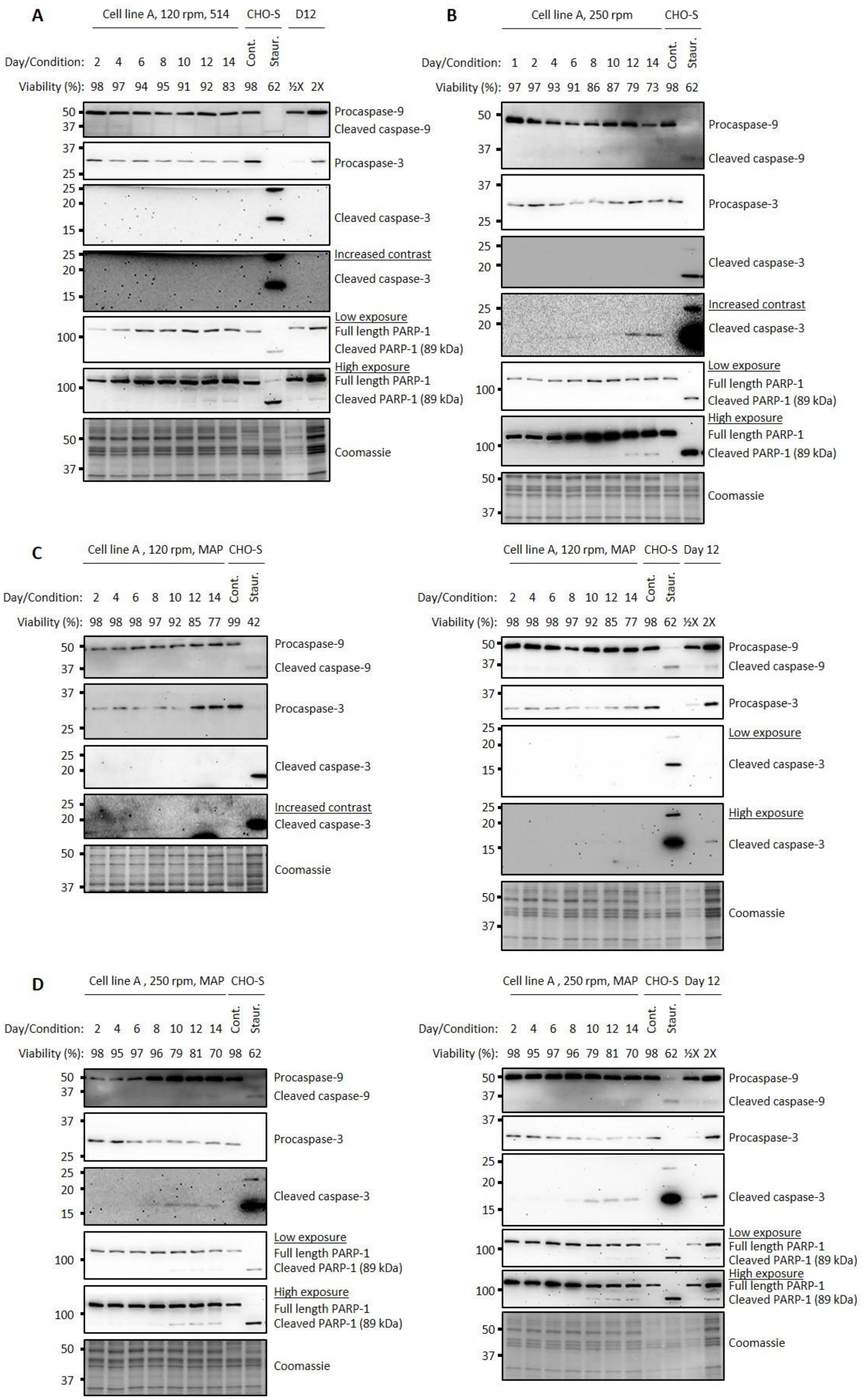
A
Cell line A, 120 rpm, 514
CHO-S
D12
Day/Condition: 2 4 6 8 10 12 14 Cont. Staur. ½X 2X
Viability (%): 98 97 94 95 91 92 83 98 62
Procaspase-9
Cleaved caspase-9
Procaspase-3
Cleaved caspase-3
Increased contrast
Cleaved caspase-3
Low exposure
Full length PARP-1
Cleaved PARP-1 (89 kDa)
High exposure
Full length PARP-1
Cleaved PARP-1 (89 kDa)
Coomassie
B
Cell line A, 250 rpm
CHO-S
Day/Condition: 1 2 4 6 8 10 12 14 Cont. Staur.
Viability (%): 97 97 93 91 86 87 79 73 98 62
Procaspase-9
Cleaved caspase-9
Procaspase-3
Cleaved caspase-3
Increased contrast
Cleaved caspase-3
Low exposure
Full length PARP-1
Cleaved PARP-1 (89 kDa)
High exposure
Full length PARP-1
Cleaved PARP-1 (89 kDa)
Coomassie
C
Cell line A , 120 rpm, MAP
CHO-S
Day/Condition: 2 4 6 8 10 12 14 Cont. Staur.
Viability (%): 98 98 98 97 92 85 77 99 42
Procaspase-9
Cleaved caspase-9
Procaspase-3
Cleaved caspase-3
Increased contrast
Cleaved caspase-3
Coomassie
Cell line A, 120 rpm, MAP
CHO-S
Day 12
Day/Condition: 2 4 6 8 10 12 14 Cont. Staur. ½X 2X
Viability (%): 98 98 98 97 92 85 77 98 62
Procaspase-9
Cleaved caspase-9
Procaspase-3
Low exposure
Cleaved caspase-3
High exposure
Cleaved caspase-3
Coomassie
D
Cell line A , 250 rpm, MAP
CHO-S
Day/Condition: 2 4 6 8 10 12 14 Cont. Staur.
Viability (%): 98 95 97 96 79 81 70 98 62
Procaspase-9
Cleaved caspase-9
Procaspase-3
Cleaved caspase-3
Low exposure
Full length PARP-1
Cleaved PARP-1 (89 kDa)
High exposure
Full length PARP-1
Cleaved PARP-1 (89 kDa)
Coomassie
Cell line A , 250 rpm, MAP
CHO-S
Day 12
Day/Condition: 2 4 6 8 10 12 14 Cont. Staur. ½X 2X
Viability (%): 98 95 97 96 79 81 70 98 62
Procaspase-9
Cleaved caspase-9
Procaspase-3
Cleaved caspase-3
Low exposure
Full length PARP-1
Cleaved PARP-1 (89 kDa)
High exposure
Full length PARP-1
Cleaved PARP-1 (89 kDa)
Coomassie

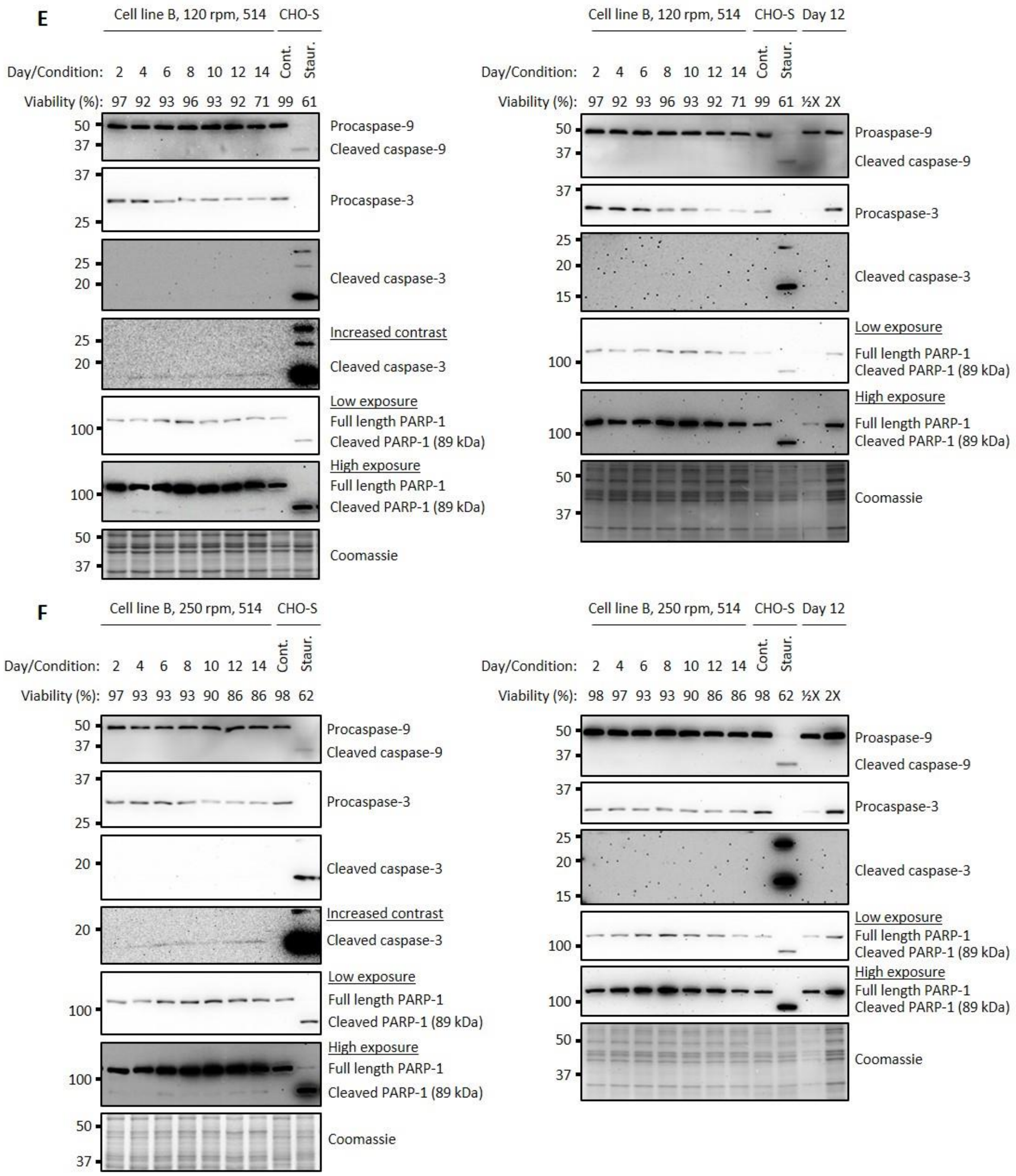

Supplementary Figure S2: Apoptosis is only detectable in cell line A at high impeller speeds. Western blot analysis of apoptotic cleaved caspases and PARP-1 under different conditions; technical replicate of Fig. 1 (A; cell line A, 120 rpm, 514 media); technical replicate of Fig. 6 (B; cell line A, 250 rpm, 514 media); cell line A, 120 rpm, MAP media (C, two technical replicates); cell line A, 250 rpm, MAP media (D, two technical replicates); cell line B, 120 rpm, 514 media (E, two technical replicates); cell line B, 250 rpm, 514 media (F, two technical replicates). Whole cell lysate was separated using SDS-PAGE gel electrophoresis. Cont. = DMSO vehicle control, Staur. = 24 hour 2 µM staurosporine treatment.

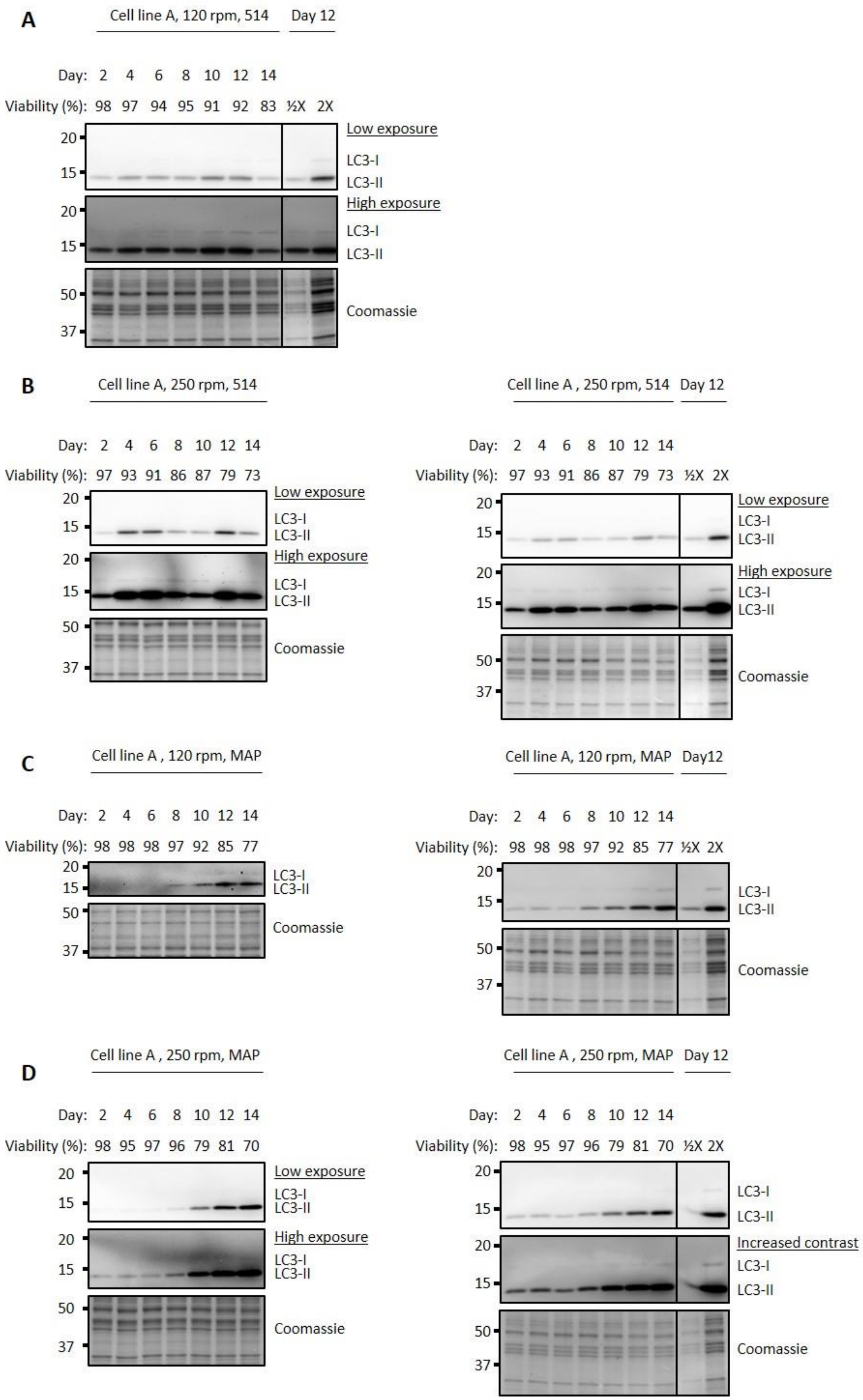
A
Cell line A, 120 rpm, 514
Day 12
Day: 2 4 6 8 10 12 14
Viability (%): 98 97 94 95 91 92 83 ½X 2X
Low exposure
LC3-I
LC3-II
High exposure
LC3-I
LC3-II
Coomassie
B
Cell line A, 250 rpm, 514
Day: 2 4 6 8 10 12 14
Viability (%): 97 93 91 86 87 79 73
Low exposure
LC3-I
LC3-II
High exposure
LC3-I
LC3-II
Coomassie
Cell line A , 250 rpm, 514
Day 12
Day: 2 4 6 8 10 12 14
Viability (%): 97 93 91 86 87 79 73 ½X 2X
Low exposure
LC3-I
LC3-II
High exposure
LC3-I
LC3-II
Coomassie
C
Cell line A , 120 rpm, MAP
Day: 2 4 6 8 10 12 14
Viability (%): 98 98 98 97 92 85 77
LC3-I
LC3-II
Coomassie
Cell line A, 120 rpm, MAP
Day12
Day: 2 4 6 8 10 12 14
Viability (%): 98 98 98 97 92 85 77 ½X 2X
LC3-I
LC3-II
Coomassie
D
Cell line A , 250 rpm, MAP
Day: 2 4 6 8 10 12 14
Viability (%): 98 95 97 96 79 81 70
Low exposure
LC3-I
LC3-II
High exposure
LC3-I
LC3-II
Coomassie
Cell line A , 250 rpm, MAP
Day 12
Day: 2 4 6 8 10 12 14
Viability (%): 98 95 97 96 79 81 70 ½X 2X
LC3-I
LC3-II
Increased contrast
LC3-I
LC3-II
Coomassie

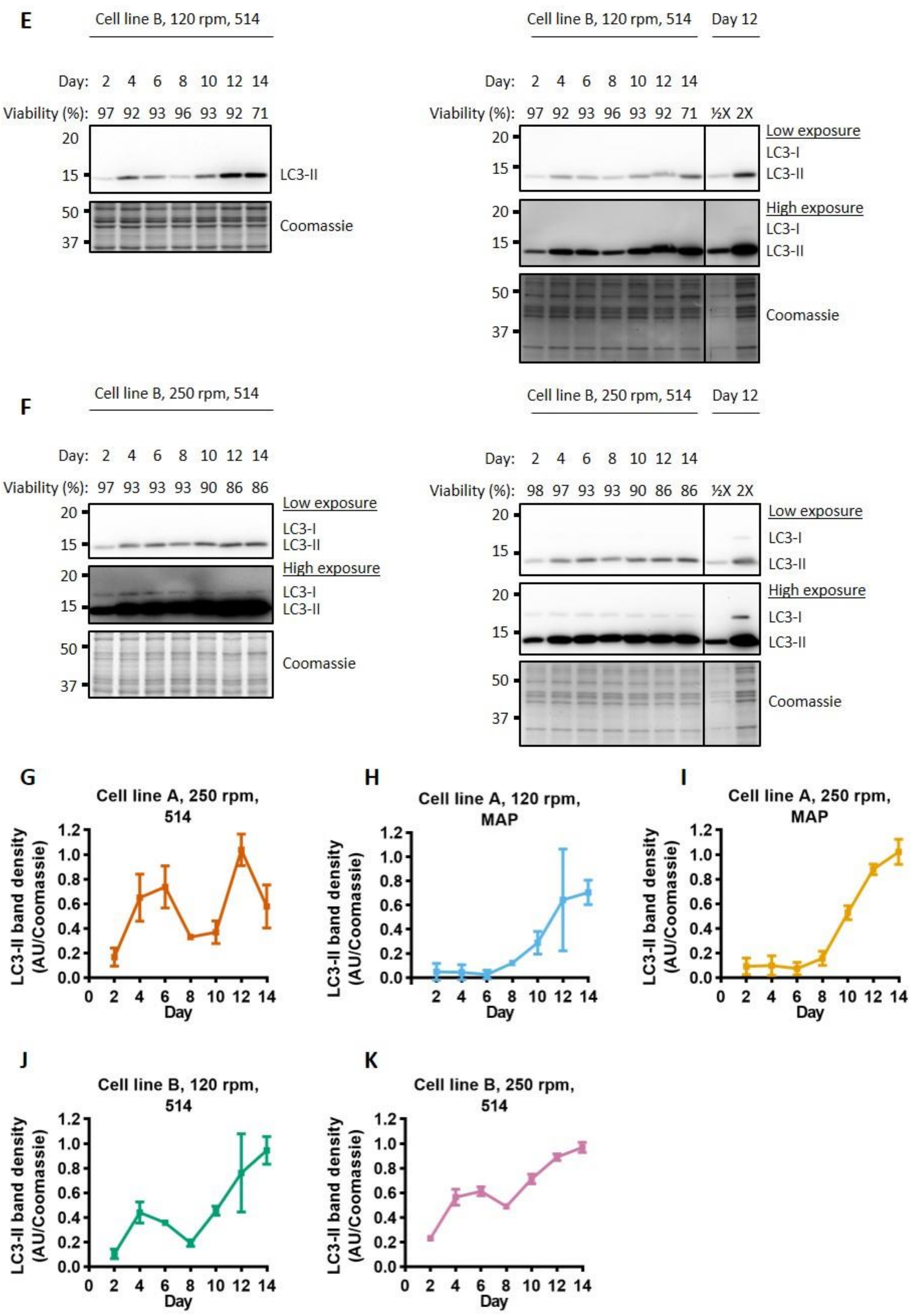

Supplementary Figure S3: Autophagy marker increases depending on the cell type and media. Western blot analysis & quantification of the autophagosome marker LC3-II under different conditions; technical replicate of Fig. 2 (A; cell line A, 120 rpm, 514 media); cell line A, 250 rpm, 514 media (B, G, two technical replicates normalised to day 12); cell line A, 120 rpm, MAP media (C, H, two technical replicates normalised to day 12); cell line A,

250 rpm, MAP media (D, I, two technical replicates normalised to day 14); cell line B, 120 rpm, 514 media (E, J, two technical replicates normalised to day 14); cell line B, 250 rpm, 514 media (F, K, two technical replicates normalised to day 14). Whole cell lysate was separated using SDS-PAGE gel electrophoresis. Images have been cropped from the same western blot or Coomassie-stained gel. Error bars represent ±SD of two technical replicates.

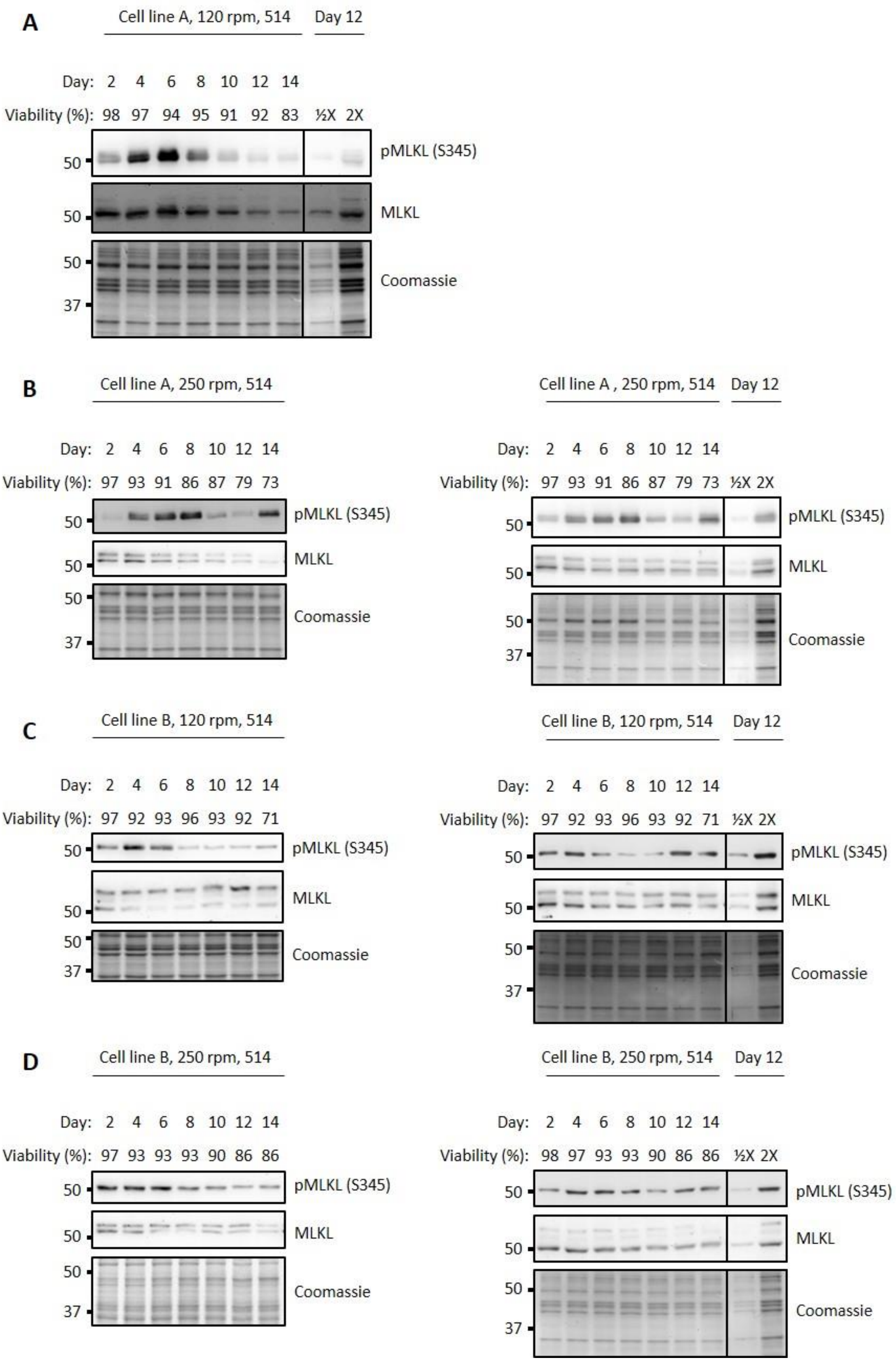
A
Cell line A, 120 rpm, 514
Day 12
Day: 2 4 6 8 10 12 14
Viability (%): 98 97 94 95 91 92 83 ½X 2X
50
pMLKL (S345)
MLKL
37
Coomassie
B
Cell line A, 250 rpm, 514
Day: 2 4 6 8 10 12 14
Viability (%): 97 93 91 86 87 79 73
Cell line A , 250 rpm, 514
Day 12
Day: 2 4 6 8 10 12 14
Viability (%): 97 93 91 86 87 79 73 ½X 2X
C
Cell line B, 120 rpm, 514
Day: 2 4 6 8 10 12 14
Viability (%): 97 92 93 96 93 92 71
Cell line B, 120 rpm, 514
Day 12
Day: 2 4 6 8 10 12 14
Viability (%): 97 92 93 96 93 92 71 ½X 2X
D
Cell line B, 250 rpm, 514
Day: 2 4 6 8 10 12 14
Viability (%): 97 93 93 93 90 86 86
Cell line B, 250 rpm, 514
Day 12
Day: 2 4 6 8 10 12 14
Viability (%): 98 97 93 93 90 86 86 ½X 2X

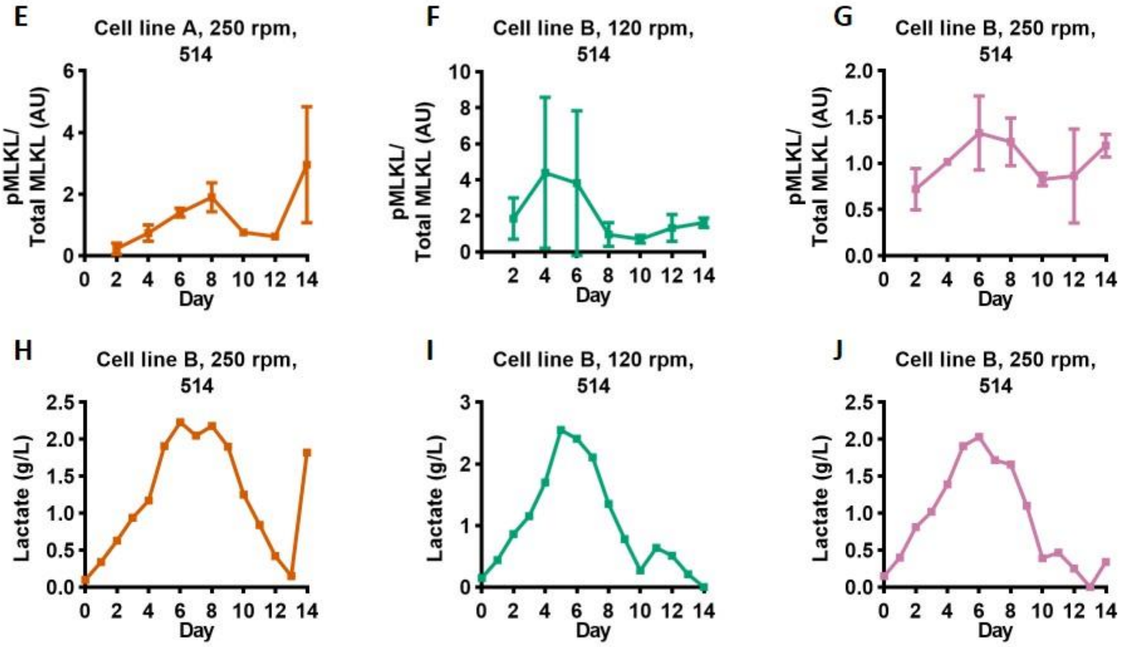

Supplementary Figure S4: Necroptosis markers correlate with lactate levels across multiple conditions. Western blot analysis & quantification of the necroptosis marker pMLKL under different conditions; technical replicate of Fig. 3 (A; cell line A, 120 rpm, 514 media); cell line A, 250 rpm, 514 media (B, E, two technical replicates normalised to day 8); cell line B, 120 rpm, 514 media (C, F, two technical replicates normalised to day 14); cell line B, 250 rpm, 514 media (D, G, two technical replicates normalised to day 4). Lactate levels measured in the media (H-J). Whole cell lysate was separated using SDS-PAGE gel electrophoresis. Images have been cropped from the same western blot or Coomassie-stained gel. Error bars represent ±SD of two technical replicates.

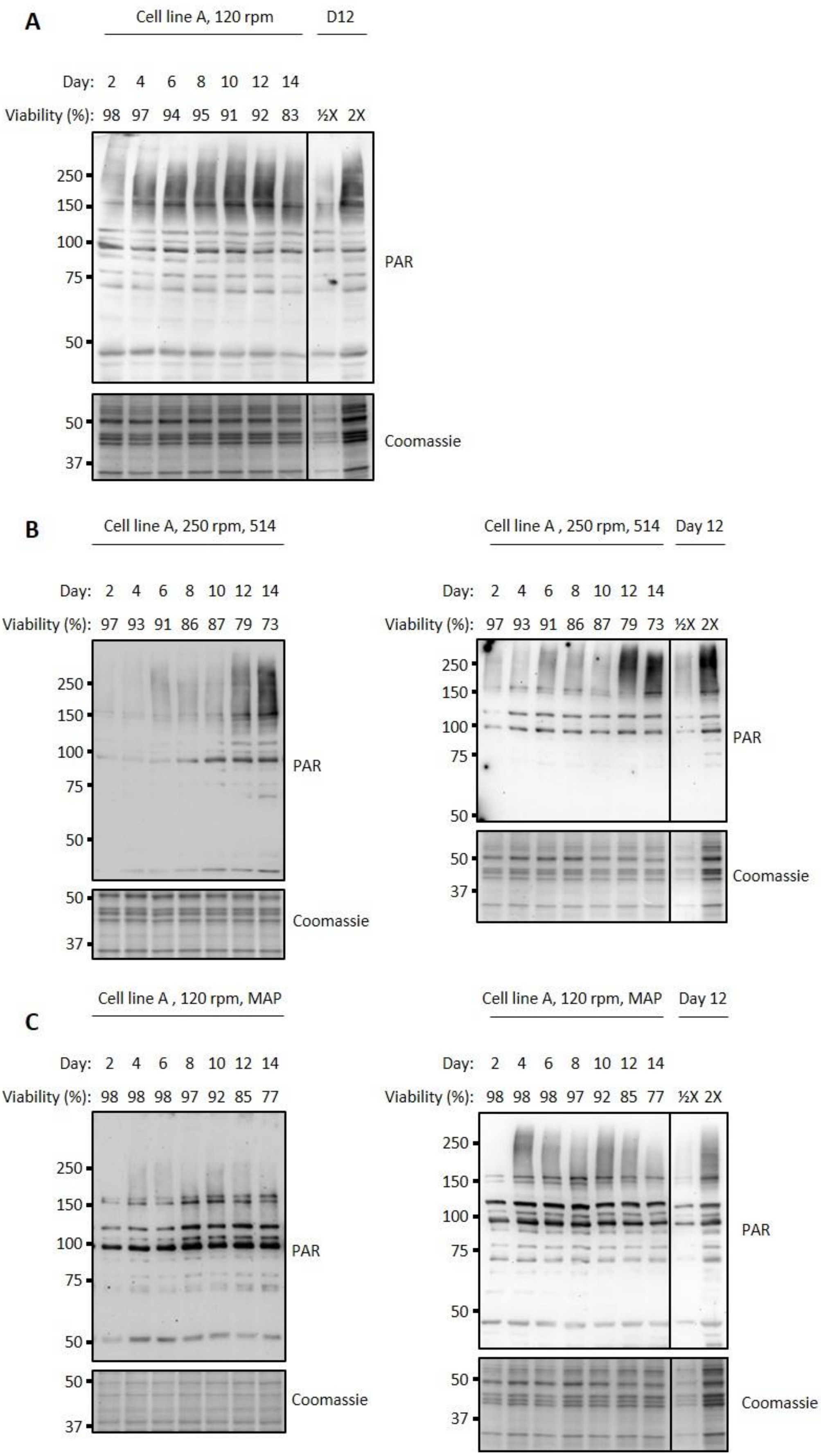
A
Cell line A, 120 rpm
D12
Day: 2 4 6 8 10 12 14
Viability (%): 98 97 94 95 91 92 83 ½X 2X
250
150
100
75
50
PAR
50
37
Coomassie
B
Cell line A, 250 rpm, 514
Day: 2 4 6 8 10 12 14
Viability (%): 97 93 91 86 87 79 73
250
150
100
75
50
PAR
50
37
Coomassie
Cell line A , 250 rpm, 514
Day 12
Day: 2 4 6 8 10 12 14
Viability (%): 97 93 91 86 87 79 73 ½X 2X
250
150
100
75
50
PAR
50
37
Coomassie
C
Cell line A , 120 rpm, MAP
Day: 2 4 6 8 10 12 14
Viability (%): 98 98 98 97 92 85 77
250
150
100
75
50
PAR
50
37
Coomassie
Cell line A, 120 rpm, MAP
Day 12
Day: 2 4 6 8 10 12 14
Viability (%): 98 98 98 97 92 85 77 ½X 2X
250
150
100
75
50
PAR
50
37
Coomassie

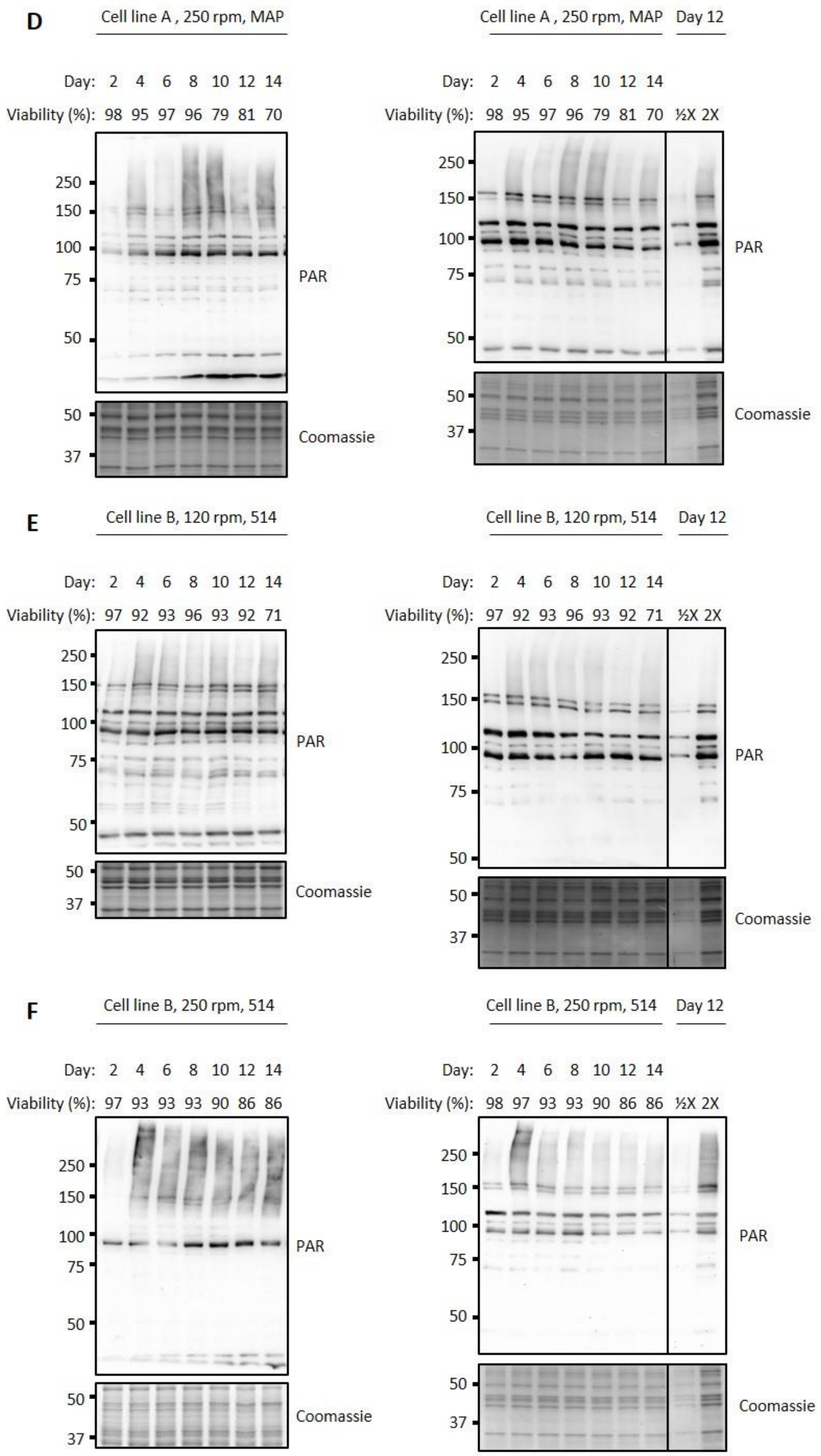
D
Cell line A , 250 rpm, MAP
Day: 2 4 6 8 10 12 14
Viability (%): 98 95 97 96 79 81 70
250
150
100
75
50
PAR
50
37
Coomassie
Cell line A , 250 rpm, MAP
Day 12
Day: 2 4 6 8 10 12 14
Viability (%): 98 95 97 96 79 81 70 ½X 2X
250
150
100
75
50
PAR
50
37
Coomassie
E
Cell line B, 120 rpm, 514
Day: 2 4 6 8 10 12 14
Viability (%): 97 92 93 96 93 92 71
250
150
100
75
50
PAR
50
37
Coomassie
Cell line B, 120 rpm, 514
Day 12
Day: 2 4 6 8 10 12 14
Viability (%): 97 92 93 96 93 92 71 ½X 2X
250
150
100
75
50
PAR
50
37
Coomassie
F
Cell line B, 250 rpm, 514
Day: 2 4 6 8 10 12 14
Viability (%): 97 93 93 93 90 86 86
250
150
100
75
50
PAR
50
37
Coomassie
Cell line B, 250 rpm, 514
Day 12
Day: 2 4 6 8 10 12 14
Viability (%): 98 97 93 93 90 86 86 ½X 2X
250
150
100
75
50
PAR
50
37
Coomassie

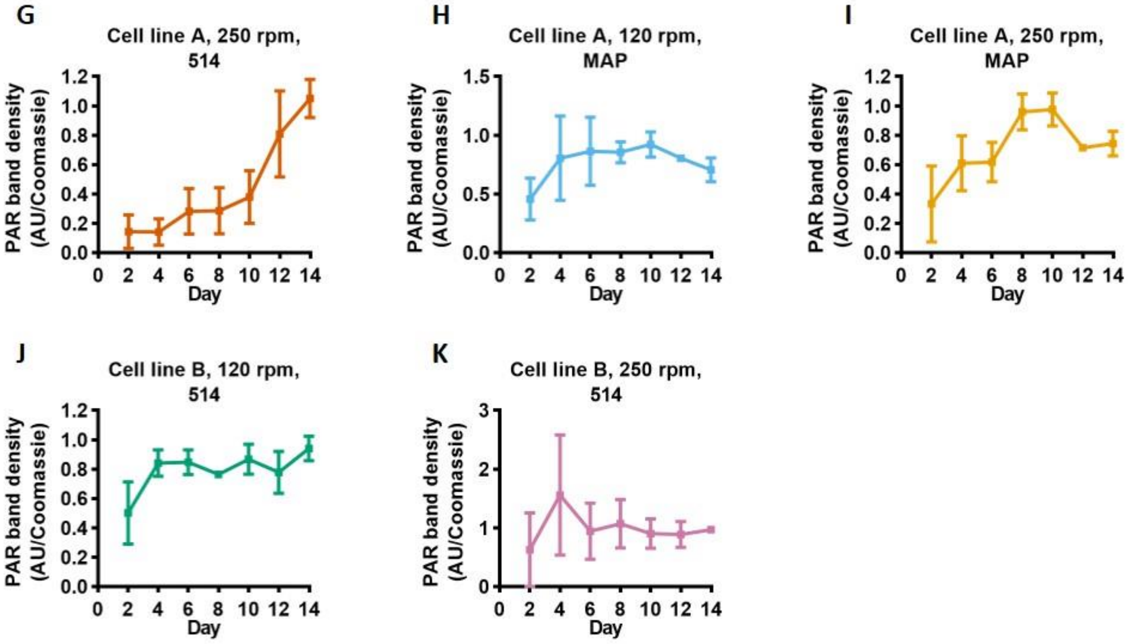

Supplementary Figure S5: PAR accumulation is greater at higher impeller speeds in cell line A. Western blot analysis & quantification of the parthanatos marker PAR under different conditions; technical replicate of Fig. 4 (A; cell line A, 120 rpm, 514 media); cell line A, 250 rpm, 514 media (B, G, two technical replicates normalised to day 14); cell line A, 120 rpm, MAP media (C, H, two technical replicates normalised to day 14); cell line A, 250 rpm, MAP media (D, I, two technical replicates normalised to day 8); cell line B, 120 rpm, 514 media (E, J, two technical replicates normalised to day 4); cell line B, 250 rpm, 514 media (F, K, two technical replicates normalised to day 14). Whole cell lysate was separated using SDS-PAGE gel electrophoresis. Images have been cropped from the same western blot or Coomassie-stained gel. Error bars represent ±SD of two technical replicates.

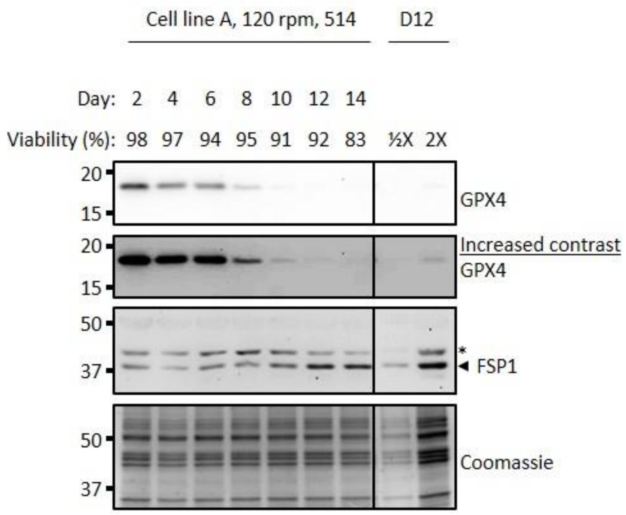

Supplementary Figure S6: Markers of ferroptosis are associated with a decline in viability. Technical replicate of Fig. 5; western blot analysis of ferroptosis suppression enzymes, GPX4 and FSP1. Whole cell lysate was separated using SDS-PAGE gel electrophoresis. Images have been cropped from the same western blot or Coomassie-stained gel. * = Non-specific band

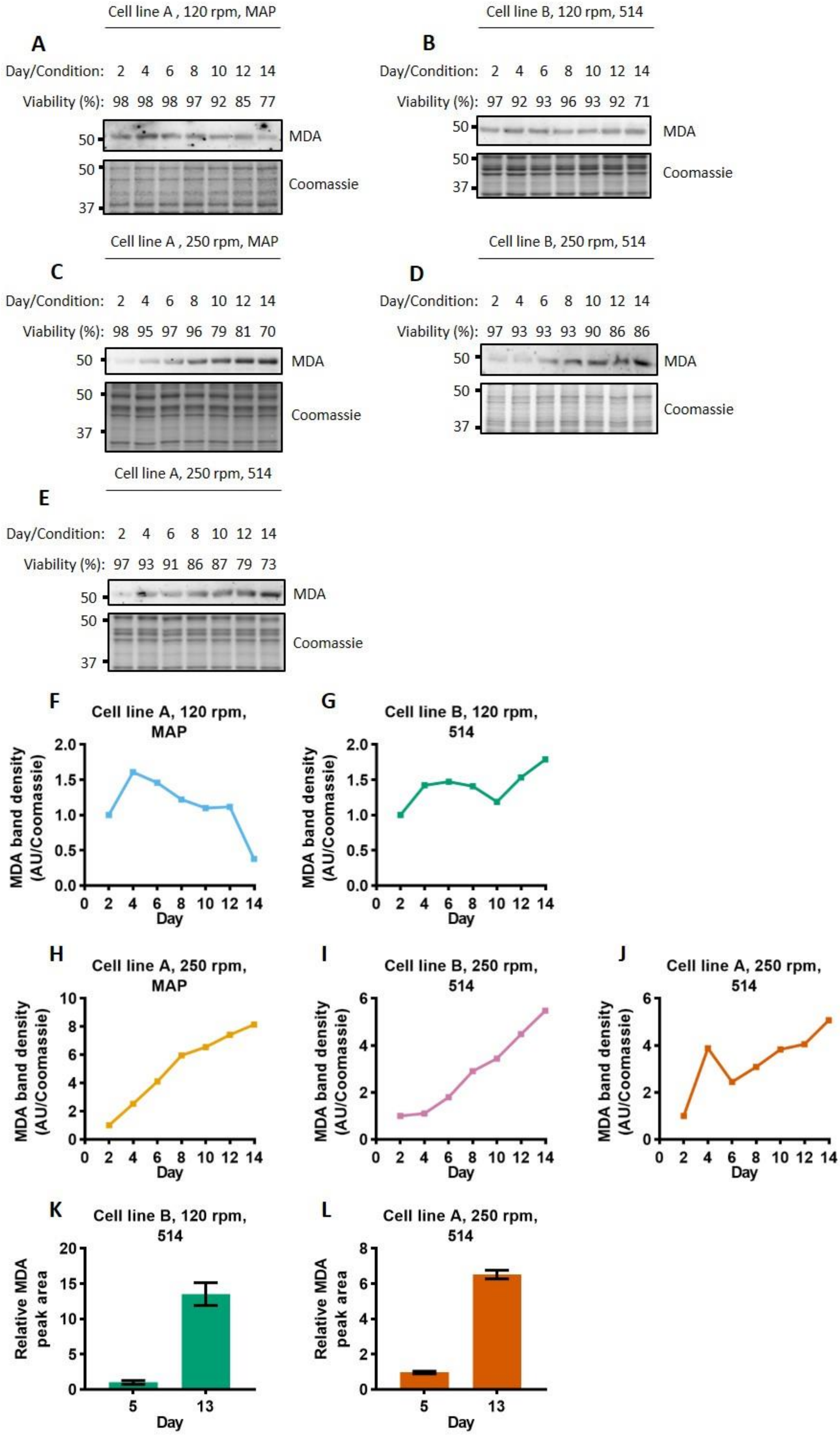

Cell line A , 120 rpm, MAP
A
Day/Condition: 2 4 6 8 10 12 14
Viability (%): 98 98 98 97 92 85 77
50
MDA
50
37
Coomassie
Cell line B, 120 rpm, 514
B
Day/Condition: 2 4 6 8 10 12 14
Viability (%): 97 92 93 96 93 92 71
50
MDA
50
37
Coomassie
Cell line A , 250 rpm, MAP
C
Day/Condition: 2 4 6 8 10 12 14
Viability (%): 98 95 97 96 79 81 70
50
MDA
50
37
Coomassie
Cell line B, 250 rpm, 514
D
Day/Condition: 2 4 6 8 10 12 14
Viability (%): 97 93 93 93 90 86 86
50
MDA
50
37
Coomassie
Cell line A, 250 rpm, 514
E
Day/Condition: 2 4 6 8 10 12 14
Viability (%): 97 93 91 86 87 79 73
50
MDA
50
37
Coomassie
F
Cell line A, 120 rpm, MAP
MDA band density (AU/Coomassie)
Day
G
Cell line B, 120 rpm, 514
MDA band density (AU/Coomassie)
Day
H
Cell line A, 250 rpm, MAP
MDA band density (AU/Coomassie)
Day
I
Cell line B, 250 rpm, 514
MDA band density (AU/Coomassie)
Day
J
Cell line A, 250 rpm, 514
MDA band density (AU/Coomassie)
Day
K
Cell line B, 120 rpm, 514
Relative MDA peak area
Day
L
Cell line A, 250 rpm, 514
Relative MDA peak area
Day

Supplementary Figure S7: Lipid peroxidation marker increases with higher impeller speeds. Western blot analysis & quantification of the lipid peroxidation marker MDA (due to discontinuation of the antibody only one technical replicate was performed) under different conditions; cell line A, 120 rpm, MAP media (A & F); cell line B, 120 rpm, 514 media (B & G); cell line A, 250 rpm, MAP media (C & H); cell line B, 250 rpm, 514 media (D & I); cell line A, 250 rpm, 514 media (E & J). Whole cell lysate was separated using SDS-PAGE gel electrophoresis. Quantification of MDA using LC-MS; cell line B, 120 rpm, 514 media (K); cell line A, 250 rpm, 514 media (L). Error bars represent ±SD of three technical replicates.

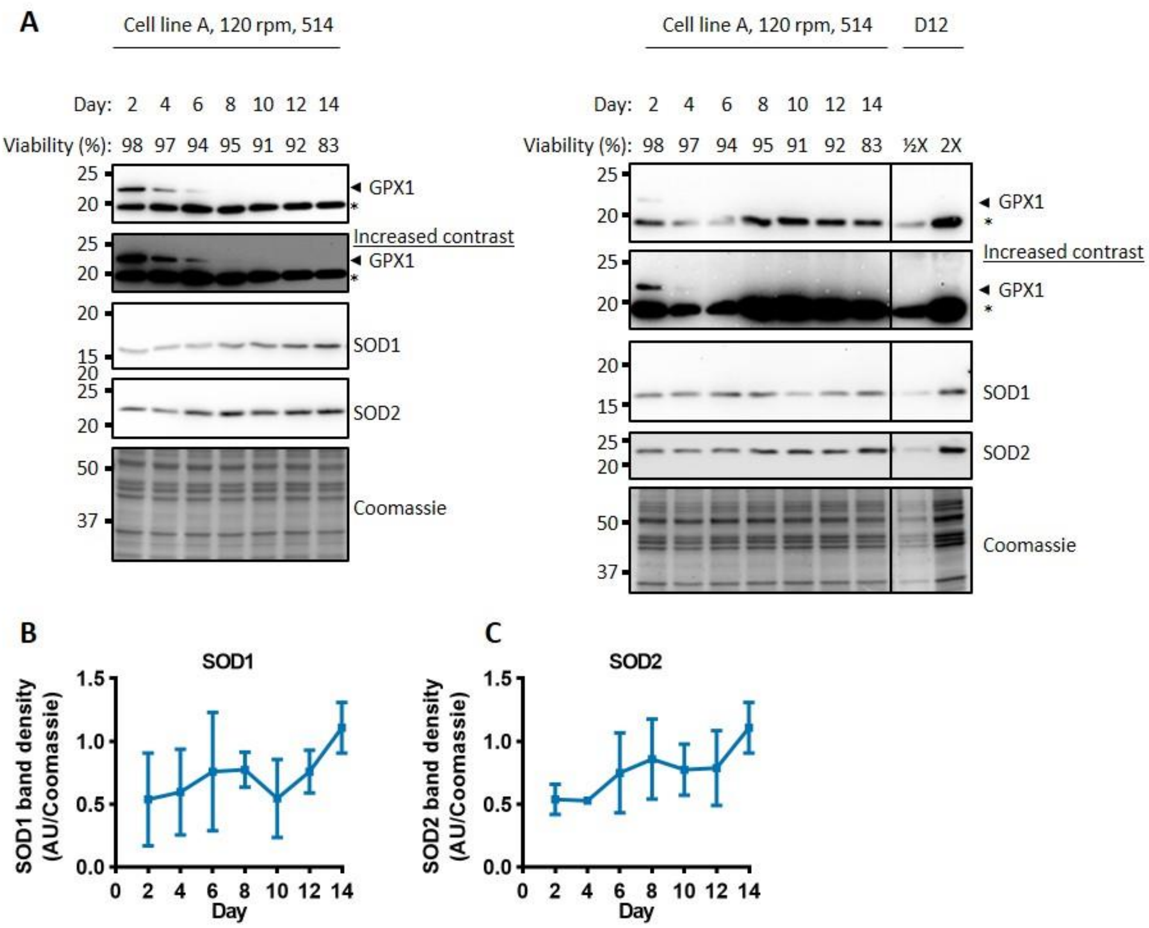

Supplementary Figure S8: Antioxidant enzyme levels during fed-batch culture in cell line A under standard conditions. Western blot analysis of the antioxidant enzymes GPX1, SOD1, SOD2 (two technical replicates) (A), and their quantification (C & D). Whole cell lysate was separated using SDS-PAGE gel electrophoresis. Images have been cropped from the same western blot or Coomassie-stained gel. Error bars represent ±SD of two technical replicates normalised to day 14. * = non-specific band.

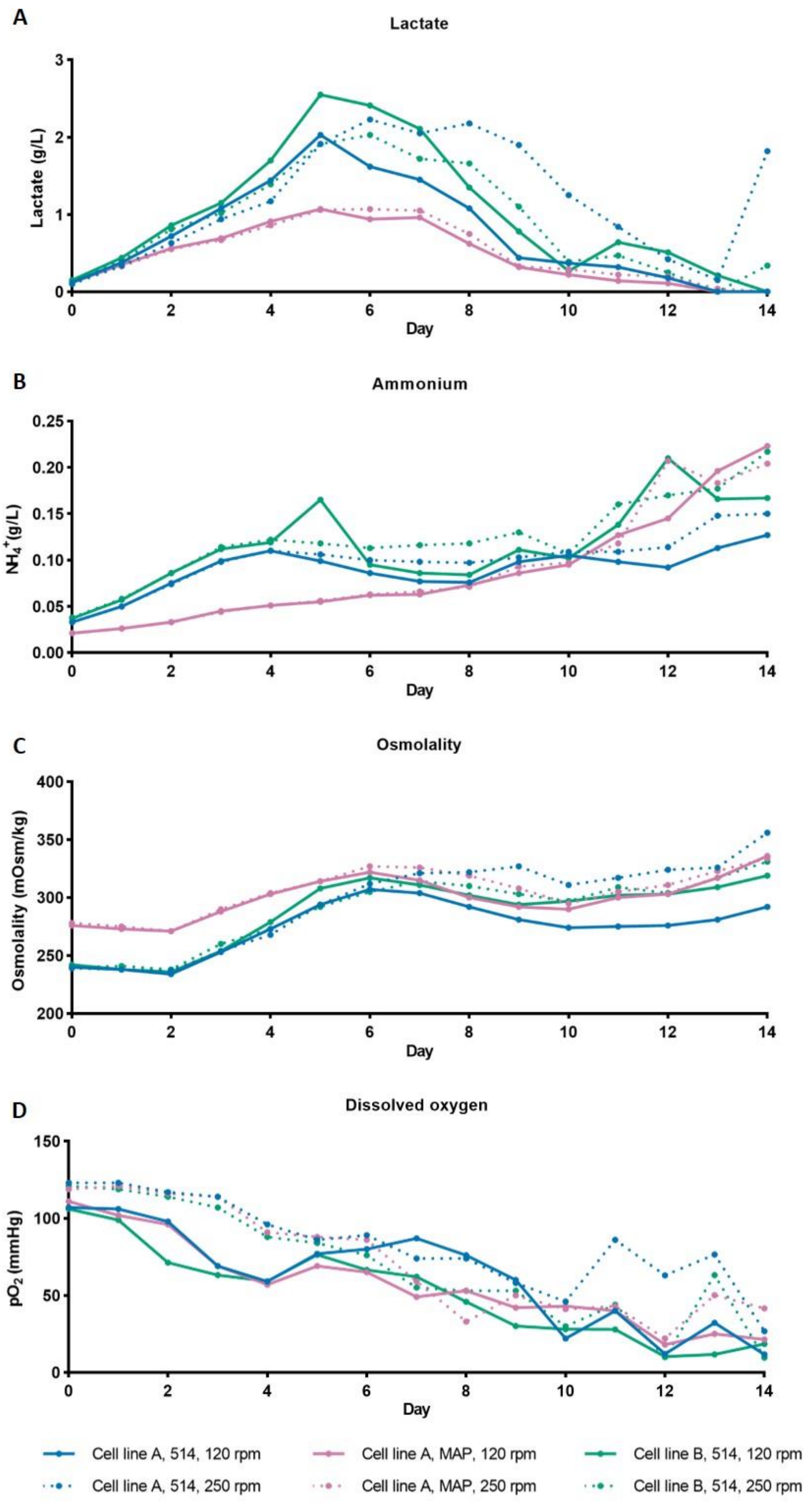

Supplementary Figure S9: Lactate, ammonium and osmolality changes during fed-batch culture. Profiles of waste metabolites and osmolality of cells grown in 10 litre bioreactors under fed-batch conditions; lactate (A), ammonium (B), osmolality (C), dissolved oxygen (D).

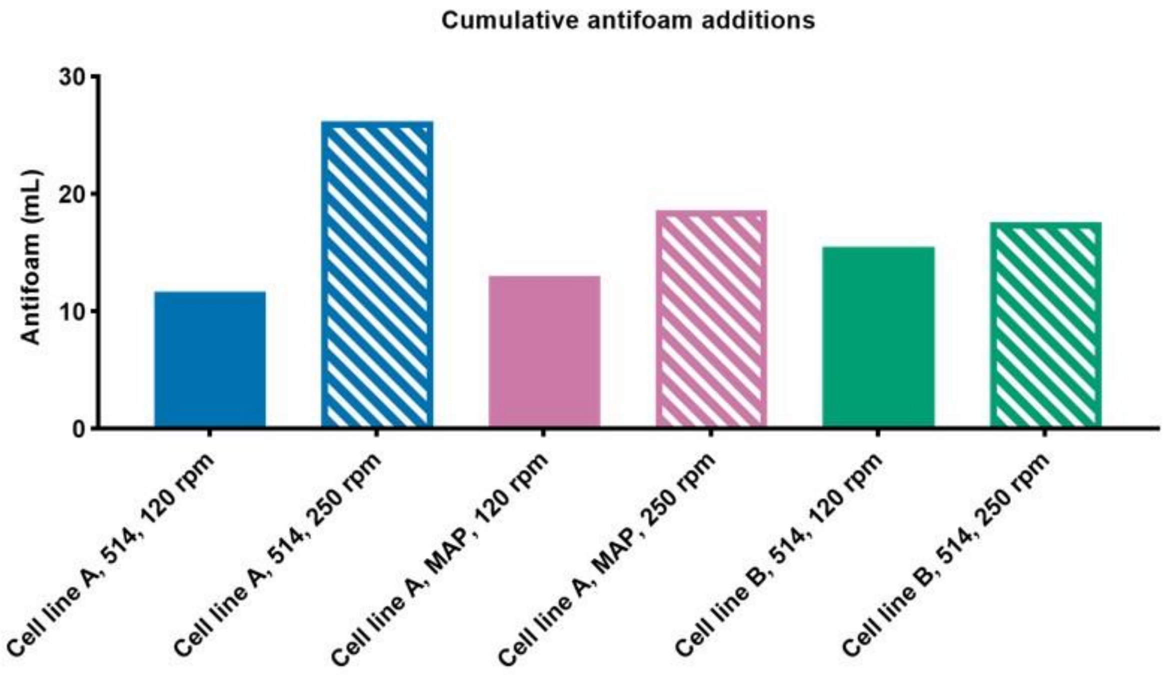

Supplementary Figure S10: Cumulative antifoam additions of cells grown in fed-batch culture in the 10-litre bioreactors.
